# Nanostructured supramolecular networks from self-assembled diamondoid molecules under ultracold conditions†

**DOI:** 10.1039/d3cp02367b

**Published:** 2023-06-13

**Authors:** Marija Alešković, Florian Küstner, Roman Messner, Florian Lackner, Wolfgang E. Ernst, Marina Šekutor

**Affiliations:** a Department of Organic Chemistry and Biochemistry, Ruđer Bošković Institute Bijenička cesta 54 10 000 Zagreb Croatia msekutor@irb.hr; b Institute of Experimental Physics, Graz University of Technology Petersgasse 16 8010 Graz Austria wolfgang.ernst@tugraz.at florian.lackner@tugraz.at

## Abstract

Diamondoid molecules and their derivatives have attracted attention as fascinating building blocks for advanced functional materials. Depending on the balance between hydrogen bonds and London dispersion interactions, they can self-organize in different cluster structures with functional groups tailored for various applications. Here, we present a new approach to supramolecular aggregation where self-assembly of diamondoid acids and alcohols in the ultracold environment of superfluid helium nanodroplets (HNDs) was analyzed by a combination of time-of-flight mass spectrometry and computational tools. Experimentally observed magic numbers of the assembled cluster sizes were successfully identified and computed cluster structures gave valuable insights into a different conglomeration mode when compared to previously explored less-polar diamondoid derivatives. We have confirmed that functional groups acting as good hydrogen bond donors completely take over the self-organization process, resulting in fascinating pair-wise or cyclic supramolecular assemblies. Particularly noteworthy is that mono- and bis-substituted diamondoid derivatives of both series engage in completely different modes of action, which is reflected in differing non-covalent cluster geometries. Additionally, formed cyclic clusters with a polar cavity in the center and a non-polar diamondoid outer layer can be of high interest in porous material design and provide insights into the structural requirements needed to produce bulk materials with desired properties.

## Abstract 2

Both diamondoid acids and alcohols form distinct supramolecular networks in helium nanodroplets.

## Graphical abstract

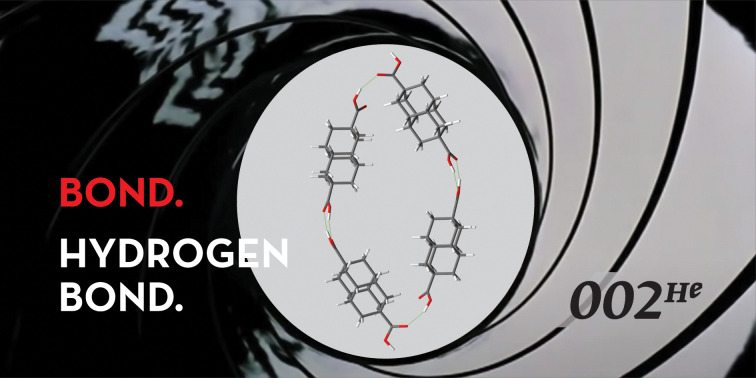

## Introduction

Diamondoids,^1,2^ cage-like hydrocarbons that are nanometer sized and naturally occurring,^3^ are very rich in C–H bonds and can act as good London dispersion (LD)^4^ energy donors. They share with their diamond counterpart some excellent properties like thermal stability and rigidity. Through selective chemical functionalization their derivatives may serve for various applications in materials science and biomedical areas.^5^ Typically, diamondoid building blocks are tailored using the means of organic synthesis and then individual scaffolds are left to form, in a best case scenario, superstructures of desired properties. However, to truly achieve predictable and controllable self-assembly, one needs to understand the underlying intermolecular binding modes. Our goal was to study fundamental aspects of the binding processes in self-organized nanosized networks of diamondoid derivatives under largely unperturbed conditions. Especially, the investigation of the interplay between hydrogen bonding and dispersion attraction effects requires the use of the least interacting non-polar environment possible. To this end we turned to helium as a solvent.

The highly non-polar medium of helium nanodroplets (HNDs) offers an opportunity to non-disruptively study weak intermolecular interactions.^6–14^ By expanding gaseous helium in a high vacuum at cryogenic temperatures,^15,16^ one obtains helium aggregates suitable for trapping introduced atoms and molecules. Cluster formation is a favorable process since the binding energy of aggregation can be dissipated through the evaporation of helium atoms. Some recent examples of studied molecular clusters in HNDs include (V_2_O_5_)_*n*_,^17^ weakly bound alkali triplet-dimers^18^ and quartet-trimers,^19^ diamondoid molecules like adamantane^20–22^ and diamantane,^23^ as well as their ether derivatives,^24^*etc.*

On the other hand, introduction of hydrogen bonding in a HND cluster system is expected to provide means for spatial control of cluster self-organization and thereby offer a way to design supramolecular assemblies with the exact and repeatable arrangement of its constituent molecules. Hydrogen bonds are present in practically all biological and chemical processes and are essential for the chemistry of life, from the structural and functional features of a simple water molecule to the behavior of complex molecules such as proteins or DNA. According to the latest IUPAC recommendation, the hydrogen bond is considered to be an attractive interaction between a hydrogen atom from a molecule or a molecular fragment X–H in which X is more electronegative than H, and an atom or a group of atoms in the same or a different molecule, in which there is evidence of bond formation.^25^ Not surprisingly, hydrogen bonding is therefore an underlying phenomenon found in diverse areas spanning from supramolecular assemblies, organic synthesis, polymerization, medicinal chemistry, *etc.*, to name just a few. As hydrogen bonds are directional, they are often used as a strategic tool in the synthesis of novel supramolecular architectures with a goal to optimize molecular recognition processes that mimic nature's elegant designs.^26,27^ An important factor for our current considerations is that hydrogen bonding is especially favored in the non-polar environment, such as organic solvents.^28^

**Fig. 1:**
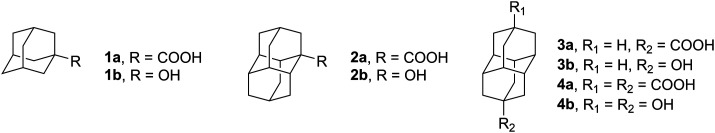
Structures of diamondoid acids 1a–4a and alcohols 1b–4b used in HND studies.

We previously investigated the influence of dispersion interactions on the self-assembly of less-polar diamondoids in HNDs.^23,24^ Going one step further, in the scope of this study we have now studied cluster formation of diamondoid acids 1a–4a and alcohols 1b–4b (Fig. 1) under HND conditions. The experimental findings were supported by a computational analysis, with special emphasis on the identified cluster sizes with large abundances, *i.e.*, the magic numbers. Our computational approach was described previously^23,24^ and applied here for the structural analysis of the clusters. To the best of our knowledge, the aggregation behavior of diamondoids in HNDs has only been studied for adamantane, diamantane, and diamondoid ethers; other diamondoids or their derivatives have remained unexplored up to now. For diamondoid acids and alcohols, molecules that possess functional groups capable of efficient hydrogen bond formation, it was expected that network assembly and stability would indeed be predominately governed by hydrogen bonding interactions. Our current work therefore had the goal to assess a possible interplay of hydrogen bonding with dispersion interactions in diamondoid acid and alcohol assemblies which were formed by clean molecule-by-molecule aggregation at low temperature inside a superfluid helium environment.

## Experimental details

### Synthesis

1-Adamantanecarboxylic acid (1a) and 1-hydroxyadamantane (1b) were purchased from Sigma-Aldrich (99% purity) and were used as received. 1-Diamantanecarboxylic acid (2a),^29^ 4-diamantanecarboxylic acid (3a),^30^ and 4,9-diamantanedicarboxylic acid (4a)^29^ as well as alcohols^31^ 1-hydroxydiamantane (2b), 4-hydroxydiamantane (3b) and 4,9-dihydroxydiamantane (4b) were synthesized according to previously published procedures.

### Helium nanodroplets

The apparatus used for the generation of the HNDs is described in detail in ref. 12,17. In short, pressurized high purity He (99.9999%) is cooled to temperatures below 20 K using a closed-cycle refrigerator (Sumitomo RDK-408D2) and expanded through a 5 μm nozzle in a high vacuum. During this process, the gaseous He condenses into small superfluid droplets. Under the expansion conditions used in the experiments (*p*_He_ = 60 bars, *T*_He_ = 11.5–12.5 K), He droplets with a mean diameter of 40 to 60 nm, consisting of about 1 × 10^6^ to 3 × 10^6^ He atoms, are formed.^15,16^ Subsequently, the beam passes a skimmer and the helium droplets pick up the desired dopant species in a separately pumped chamber. Here, we dope the droplets with diamondoid derivatives using a heated gas-pickup cell (96 °C for 1a, 200 °C for 2a, 180 °C for 3a, 45 °C for 1b, 120 °C for 2b, and 114 °C for 3b), which is connected to a heated reservoir (70 °C) *via* a precision leak valve or using a heated crucible directly in the pickup chamber (320 °C for 4a and 145 °C for 4b). The doped He droplets then enter the differentially pumped analysis chamber, where a reflectron time-of-flight mass spectrometer KAESDORF RTF50 is utilized to record the mass spectra. Upon electron impact ionization, cluster ions are expelled from the He droplets and can be detected at the corresponding mass channel. The employed emission current (*I*_em_) and ionization energy (*E*_el_) are *I*_em_ = 6.8 μA and *E*_el_ = 90 eV, respectively.

### Theoretical methods

Geometry optimizations of acids 1a, 2a and 4a and alcohols 1b–4b were performed using the Orca 5.0.3 program package^32,33^ using the B3LYP-gCP-D3(BJ)-ABC/def2-TZVPP level of theory,^34,35^ and the obtained minima were verified by frequency computations. A search for favorable cluster structures was done using the Conformer-Rotamer Ensemble Sampling Tool (CREST) based on the GFN methods^36,37^ by applying the iterative *meta*-dynamic sampling for non-covalently bound complexes, clusters or aggregates (NCI-iMTD mode). Single point computations on the identified best cluster structures were performed using the B3LYP-gCP-D3(BJ)-ABC/def2-TZVPP level of theory. The choice of the DFT method was based on our previous benchmarking of similarly bulky molecules in the HNDs.^23,24^ We found that using the D3(BJ) dispersion correction,^34,35,38^ the three-body dispersion contribution term implemented in Orca as well as the geometrical counterpoise (gCP) correction^39^ successfully accounts for subtle intermolecular interactions and mitigates the basis-set superposition errors (BSSEs), respectively. Please see the ESI† for more computational details.

## Results and discussion

In previous studies of self-organized HND aggregates consisting of diamondoid molecules such as adamantane,^20–22^ diamantane^23^ and diamondoid ethers,^24^ certain preferences for cluster abundances were found that belonged to particularly stable geometries. These observed privileged abundances were termed as magic numbers. Additionally, the intermolecular dispersion attraction between bulky hydrocarbons had a pronounced effect on supramolecular binding and cluster formation in the HNDs. We were interested in exploring how the introduction of hydrogen bond donating groups affects the aggregation of diamondoid molecules in a HND medium, unimpeded by the effect of common solvents. When the organic molecule is no longer a pure hydrocarbon but still of low polarity, like in the case of diamondoid ethers, dispersion attraction still acts as a driving force for cluster formation but hydrogen bonding between ether oxygens and trace water molecules leads to an eventual breakdown of the initial supramolecular aggregates. With this in mind, we went further up on the relative polarity scale and prepared diamondoid acids 1a–4a and alcohols 1b–4b (Fig. 1). Acid 1a and alcohol 1b consist of an adamantane scaffold, while acids 2a–4a and alcohols 2b–4b incorporate the diamantane cage carrier. In continuation, we will focus on each series of the prepared diamondoid derivatives.

### Diamondoid acids

To date, investigations of organic acids in the helium environment were mostly limited to small molecules like formic and acetic acids. The HND studies of formic acid, the simplest of organic carboxylic acids, included characterization studies of monomers and clusters of both the parent molecule^40^ and its deuterated form.^41^ Special attention was paid to its dimer which is the smallest organic prototype for a double-hydrogen-bonded system. Surprisingly, the preferred formation of an acyclic polar dimer in HNDs,^42,43^ a structure that is not a global energetic minimum, demonstrated yet again the uniqueness of this medium for the exploration of non-covalently bound clusters. Namely, instead of the double-hydrogen-bonded acid dimer structure that has two strong and equivalent O–H⋯O=C interactions and is typically found in the gas phase, the observed structure of formic acid dimers in the HNDs adopted a molecular orientation where one strong O–H⋯O=C and one relatively weak C=O⋯H–C interaction co-existed. A similar observation was also found for acetic acid dimers,^44–46^ where the preferential formation of metastable acyclic pair-wise assemblies in the HNDs was in contrast to the cyclic species usually dominant in the gas phase. It was suggested that acyclic acid clusters with their asymmetric charge distributions interact more readily with He and thereby form stable clusters with helium atoms attached.^47^ Such an influence of helium atoms on metastable dimer abundances was also recently demonstrated using molecular dynamics computations.^47^ As the acid monomer is embedded into a helium medium, the collision with the second acid molecule results in the release of excess energy through helium atom evaporation and the formed dimer is prone to keep the initial relative orientations of the acid molecules, unlike in the gas phase where the collision energy is not dispersed in such a way and the distribution of internal energies can be very broad.

**Fig. 2:**
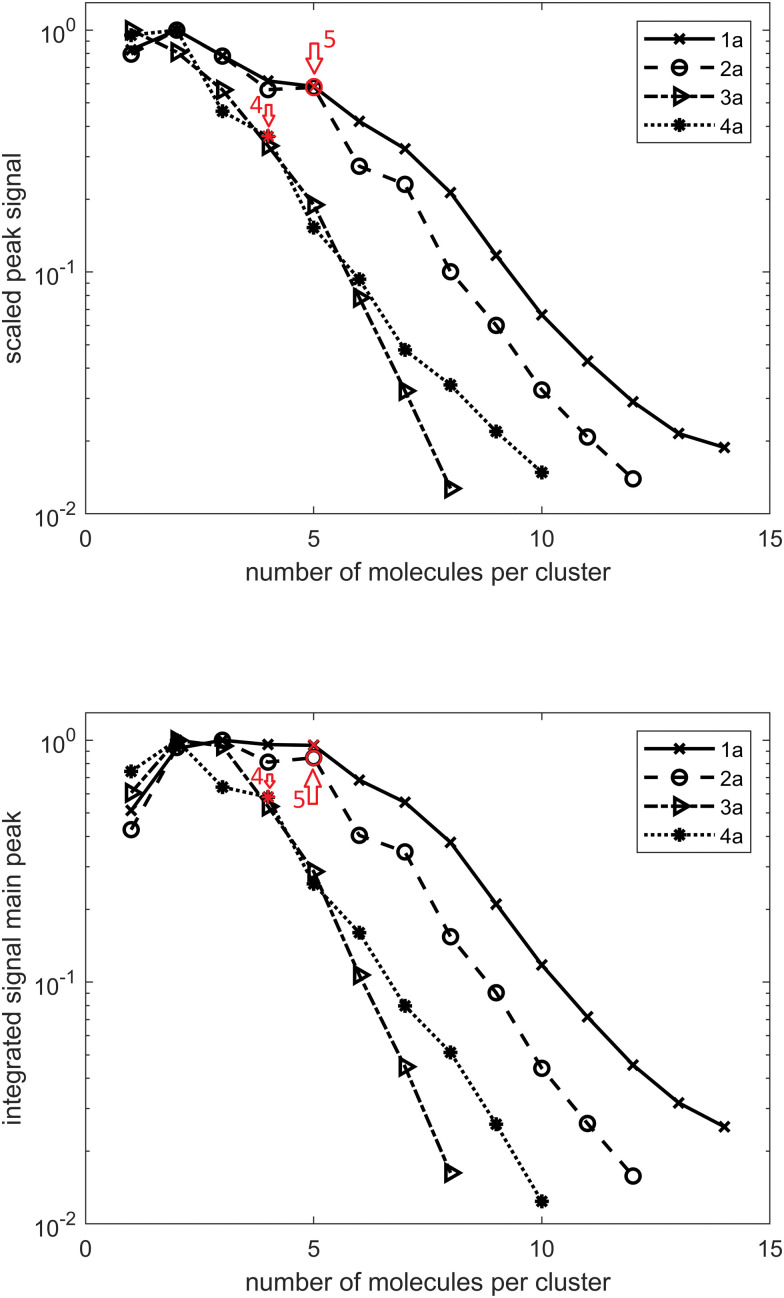
TOF signal curves for adamantane acid 1a, diamantane medial acid 2a, diamantane mono-apical acid 3a, and diamantane bis-apical diacid 4a. (a) Peak height scaled by the strongest signal of each compound. (b) Integrated peak area scaled by the largest peak area of each compound.

In the case of diamondoid acids, aside from the dimer structures, we also observed the formation of larger magic number clusters. Fig. 2(a) shows the course of the mass peak height with respect to the number of acid molecules per cluster for the investigated compounds. In panel Fig. 2(b) the integrated peak area is plotted with respect to the number of acid molecules per cluster. Both indicate that a cluster of five molecules for adamantane acid 1a and diamantane medial acid 2a is particularly stable as well as a cluster of four molecules for diamantane bis-apical diacid 4a. This is also confirmed by the spectra shown in Fig. S1 (ESI†). There seems to be no preferable cluster size for diamantane mono-apical acid 3a, for which the signal drop-off is also the strongest with an increasing cluster size, suggesting that clusters of 3a are the weakest bound.

Moreover, for every cluster size, there is a series of additional peaks at larger mass numbers. These can be assigned to a specific number of water molecules added to the cluster. In Fig. S1 (ESI†), the peak areas with respect to the number of additional water molecules are indicated, with each line scaled by its highest value. It is striking that for small clusters of 1a and 2a no additional water molecule seems to be favorable and for larger clusters this shifts to one additional water molecule. For 1a this transition is located between the cluster sizes eight and nine and for 2a it is located between the cluster sizes seven and eight. This is probably due to the stabilization of the larger cluster by the water molecule. For 4a something similar is observable for cluster sizes between six and seven; however, here the preferred number of water molecules changes from zero to two.

The computational analysis of the experimentally most abundant clusters formed by studied diamondoid acids in the HNDs, 1aCL5 (*n* = 5), 2aCL5 (*n* = 5) and 4aCL4 (*n* = 4), revealed informative details about structural organization (Fig. S3, ESI†). Building on our previous benchmarking for similar systems,^23,24^ we performed a search for viable cluster structures using CREST based on the GFN methods,^36,37^ more specifically, by applying the iterative *meta*-dynamic sampling for non-covalently bound systems (NCI-iMTD mode). After identifying the best candidates, we performed single point computations on these geometries using the B3LYP-gCP-D3(BJ)-ABC/def2-TZVPP level of theory.

The obtained interaction energies (Table S3, ESI†) for the here studied acid clusters are in line with our previously obtained values for diamantane^23^ and diamondoid ether^24^ clusters, confirming that the aggregation of diamondoid acids in HNDs is indeed an energetically favorable process. However, in contrast to the aforementioned aggregates, intermolecular LD is no longer a decisive factor for cluster aggregation; this role here goes to hydrogen bonding. For example, by observing the spatial arrangement of acid molecules in the computed adamantane 1aCL5 and medial diamantane 2aCL5 clusters (Fig. S3, ESI†), one observes the acid pair formation of an O–H⋯O=C pattern. As these magic number clusters are odd-numbered, the lone acid molecule alternates between the existing pairs and engages in a bridging interaction. Remarkably, cluster 4aCL4 possesses completely different structural features. As the parent molecule has two acid moieties substituted in the apical positions of the diamantane cage, the resulting cluster consisting of four 4a molecules readily forms a cyclic supramolecular assembly. In this cavity-forming assembly held together by hydrogen bonding, two bridges engage in interactions of a double O–H⋯O=C type while the remaining two engage in a single O–H⋯O=C interaction between the two neighboring acid groups (Fig. S3, ESI†). Thus, such structural cluster arrangement directly showcases the power of hydrogen bonding in diamondoid acid agglomeration processes and confirms that neither LD interactions between numerous good dispersion energy donor scaffolds nor solvating stabilization achieved by weak helium interactions can compete with strong hydrogen bond forming functional groups like acid moieties. Unlike small acid dimers of formic acid or acetic acid, larger diamondoid acids seem to be less influenced by the collision energy distribution upon cluster formation and one in principle observes strong O–H⋯O=C bonding patterns between the molecules. This may be due to the physicochemical nature of diamondoid acids as they consist not only of hydrogen bond forming groups but also incorporate bulky and heavy cage subunits. Note that the principle of cavity tailoring of hydrogen-bonded molecular scaffolds using relatively weak intermolecular contacts was described previously and can be a useful tool for the design of functional supramolecular assemblies.^48^

**Fig. 3:**
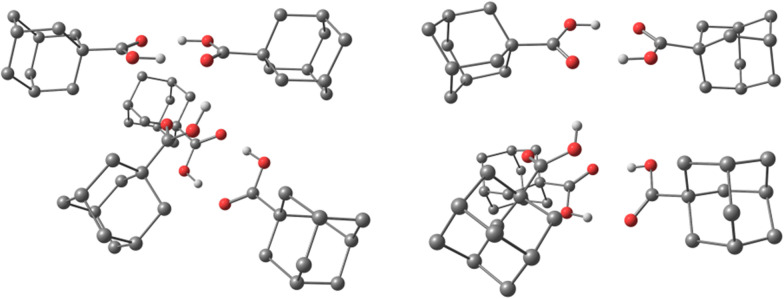
Optimized structures of 1aCL5 (left) and 1aCL5_500He (right) obtained from computations (*vide supra*). Color codes: C, grey; H, white; and O, red. Helium atoms and hydrogen atoms attached to carbon are removed for clarity.

To confirm that our computational findings are valid beyond just gas phase conditions, we performed the optimization of the cluster consisting of five 1a molecules (analogous to 1aCL5) but also having in the beginning each 1a molecule spatially isolated and surrounded by 100 He atoms. The proceeding of the optimization and the resulting cluster structure confirms that the agglomeration of diamondoid acids is indeed preferential to simple “solvation” of individual molecules with helium atoms (ESI,†1aCL5_500He). Moreover, the obtained final structure consisting of a diamondoid acid molecules core and an outer helium shell is comparable to the structure of cluster 1aCL5, with similar hydrogen bonding patterns between acid moieties observed (Fig. 3). Note also that the emerged molecules core of the final computed 1aCL5_500He structure (ESI†) contains no helium atoms at all dispersed between diamondoid subunits. This clearly demonstrates that not only does the hydrogen bonding region expectedly push out all He out of its reach of influence, but also that diamondoid cages themselves preferentially engage in the intermolecular dispersion interaction rather than in simple “solvation” with helium, in line with our previous findings.^23,24^

Concerning the number of water molecules observed for acid clusters, smaller assemblies of 1a and 2a do not favor the presence of water and when increasing the cluster size one observes the incorporation of a single water molecule (Fig. S1, ESI†). From a structural standpoint that is to be expected since the acid moieties engage in favorable intermolecular hydrogen bonding pair-wise patterns and no place for water incorporation is available. As the cluster size and the number of pairs grow, the network seems to benefit from stabilization accomplished by including additional water. In the case of 4a, larger cluster sizes are capable of incorporating even more water molecules since this diacid is capable of forming cavities of alternating polarity (Fig. S3, ESI†), which enables easier access to hydrogen bond partners, *i.e.*, one available O or OH group per molecule.

### Diamondoid alcohols

**Fig. 4:**
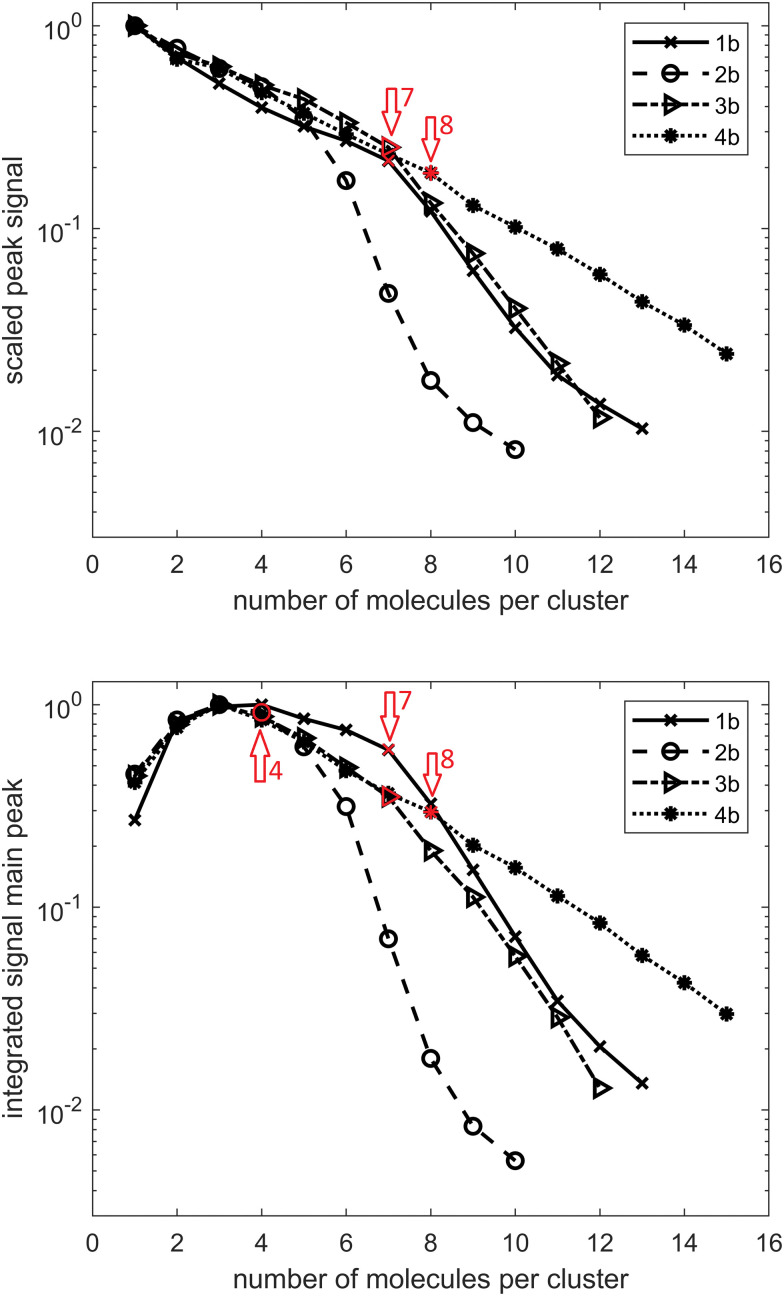
TOF signal curves for adamantane alcohol 1b, diamantane medial alcohol 2b, diamantane mono-apical alcohol 3b, and diamantane bis-apical diol 4b. (a) Peak height scaled by the strongest signal of each compound. (b) Integrated peak area scaled by the largest peak area of each compound.

Turning now our attention to alcohols, one in principle expects weaker hydrogen bonding since the alcohol O–H bond is less polarized than its acid counterpart. To date, mostly small primary, secondary and tertiary alcohols were investigated in a HND environment^49–57^ as well as several combined systems, including NaCl–methanol,^58^ fullerene and methanol or ethanol clusters,^59^ anisole–methanol,^60^ and trifluoromethoxybenzene–methanol complexes.^61^ However, as previously mentioned, no diamondoid alcohols in HNDs were characterized to date. We therefore performed a similar combined experimental and computational analysis for these alcohols in order to compare the self-assembly behaviors of the two studied classes of hydrogen bond forming groups bound to a diamondoid skeleton. One immediately notices that determination of the preferred cluster sizes for alcohols 1b–4b is not as straightforward as it was for the analogous acids. The data depicted in Fig. 4 suggest a preference of seven molecules for 1b and 3b, four molecules for 2b and eight molecules for 4b. In addition, three molecules per cluster also seem to be candidates for 3b and 4b. Similar to the acids, for 1b and 2b, one can observe a preference for larger clusters to include one water molecule. For 1b, this transition is analogous to 1a between cluster sizes of eight and nine molecules; for 2b it is between six and seven molecules. In contrast to 4a, no influence of water absorption is observed with 4b.

When analyzing the computed structures for the respective clusters of alcohols 1b–3b (Fig. S4, ESI†), it becomes apparent that a strict pair-wise directionality seen in acid clusters is missing here. Namely, the alcohol molecules in the computed structures engage in cyclic hydrogen bond-held assemblies where the OH groups are oriented towards the center of the cluster and form a cavity while the diamondoid part is exposed to the helium environment. Such supramolecular structures can therefore be compared to reverse micelles where the polar parts of the molecules are pointed inwards and form a cavity while the non-polar parts are in contact with solvent molecules of low polarity, in our case helium. It therefore comes as no surprise that no strong preference for specific magic number clusters was observed; many cyclic arrangements of this type are feasible and energetically favorable. The formation of comparable cyclic clusters was also observed previously for methanol.^62^ However, although structurally less ordered and weaker in individual hydrogen bond interactions, assemblies of this type could be of even greater practical importance than their acid counterparts since they are capable of spontaneous polar cavity formation in a highly non-polar medium. As reverse micelles are often used as efficient nano-carriers capable of targeted delivery and/or solubilization of small molecules, including drugs, production and characterization of more advanced supramolecular architectures in the HNDs is an appealing venue of investigation. For example, a two substrate system that would include doping of HNDs with both a diamondoid alcohol and a polar substrate of choice or a polar solvent molecule could provide a reverse micelle^63^ with an incorporated nano-cargo, essentially making an assembly inside the assembly (HNDs are doped with diamondoid cavity self-assemblies that are in turn doped with selected polar molecules).

Clusters of alcohols 1b–3b also demonstrate the capability of water inclusion and the process is proposed to be aided by the formed central cavity. However, note that the type of cavity present in such alcohol assemblies differs from the one found in the bis-acid cluster 4aCL4. The bis-acid molecules therefore build a cyclic structure through an alternating pattern, *i.e.*, the rim is composed of repeating diamondoid cage subunits and acid groups. Despite alternating rim polarities, such an arrangement nevertheless fosters water incorporation in the cluster since, as mentioned, the two neighboring acid group pairs of the cluster engaged only in a single O–H⋯O=C interaction and are therefore free to interact with water molecules through their remaining oxygen groups. On the other hand, diamondoid alcohol clusters that resemble a reverse micelle possess a central cavity that is polar in nature and should be capable of favorably hosting water molecules in an inclusion binding event. Lastly, a marked difference is observed for clusters of bis-alcohol 4b. Assemblies of this apically bis-substituted diamantane alcohol do not prefer to incorporate water molecules at all and the reason for that can again be elucidated from the respective cluster structure 4bCL8 (Fig. S4, ESI†). One observes that this diol does not form a central polar cavity like the mono-alcohols discussed so far but rather engages in random hydrogen bonding events with the surrounding diol molecules. The result is a poorly organized supramolecular structure that resembles more a network of randomly meshed molecules than an arranged self-assembled system. This goes to show that a feature (double substitution) beneficial for one type of non-covalent cluster formation (cavity of alternating polarity for 4a) can be detrimental for another type (random organization of 4b molecules).

## Conclusions

We studied self-assembled clusters of both diamondoid acids and alcohols in superfluid helium nanodroplets by means of mass spectrometry and found that the observed magic number abundances differ when compared to both diamondoid hydrocarbons and ethers. While hydrocarbon sphere-like molecules pack much more condensely in HNDs, diamondoid ether packing and cluster abundances are strongly influenced by individual molecular geometries as well as the presence of oxygen atoms. In contrast to these predominately dispersion governed processes, the introduction of functional groups with higher polarity, which are good hydrogen bond donors, to a diamondoid scaffold drastically changes the molecular self-organization in HNDs. Thus, the computational analysis revealed that mono-substituted diamondoid acids readily form strictly spatially directed networks built up through pair-wise intermolecular hydrogen bonding interactions, while analogous mono-alcohols generate a wide range of cyclic clusters resembling reverse micelles. Additionally, a change was observed in a cluster self-assembly mode when going from mono- to bis-derivatives for both series of compounds. Namely, diamantane bis-apical diacid 4a forms a cavity having a pattern of alternating polarity, while structurally analogous bis-alcohol 4b produces somewhat random structural patterns with no central polar cavity that was noted for all studied diamondoid mono-alcohols. The observed cavity formation and spontaneous and spatially ordered supramolecular network organization achieved with these hydrogen-bonding systems can have implications for use in porous material production, emphasizing factors to be taken into consideration when designing materials of tailor-made properties.

## Author contributions

The manuscript was written through contributions of all authors. All authors have given approval to the final version of the manuscript.

## Conflicts of interest

The authors declare no competing financial interest.

## Supplementary Material

CP-025-D3CP02367B-s001

CP-025-D3CP02367B-s002
