# Supplementary material for: Nanostructured supramolecular networks from self-assembled diamondoid molecules under ultracold conditions
Source: Phys Chem Chem Phys. 2023 Jun 13;25(27):17869–76. doi: 10.1039/d3cp02367b (PMC10336979; doi:10.1039/d3cp02367b)
Supplement: CP-025-D3CP02367B-s002 [file CP-025-D3CP02367B-s002.pdf]

Electronic Supplementary Material (ESI) for Physical Chemistry Chemical Physics.
This journal is © the Owner Societies 2023

Supporting information

Nanostructured supramolecular networks from self-assembled diamondoid molecules at ultracold conditions

Marija Alešković,[a, ‡] Florian Küstner,[b, ‡], Roman Messner,[b] Florian Lackner [b,*] Wolfgang E. Ernst,[b,*] Marina Šekutor[a,*]

[a] Department of Organic Chemistry and Biochemistry, Ruđer Bošković Institute, Bijenička cesta 54, 10 000 Zagreb, Croatia, msekutor@irb.hr

[b] Institute of Experimental Physics, Graz University of Technology, Petersgasse 16, 8010 Graz, Austria, wolfgang.ernst@tugraz.at, florian.lackner@tugraz.at

[‡]Both contributors are considered first authors.

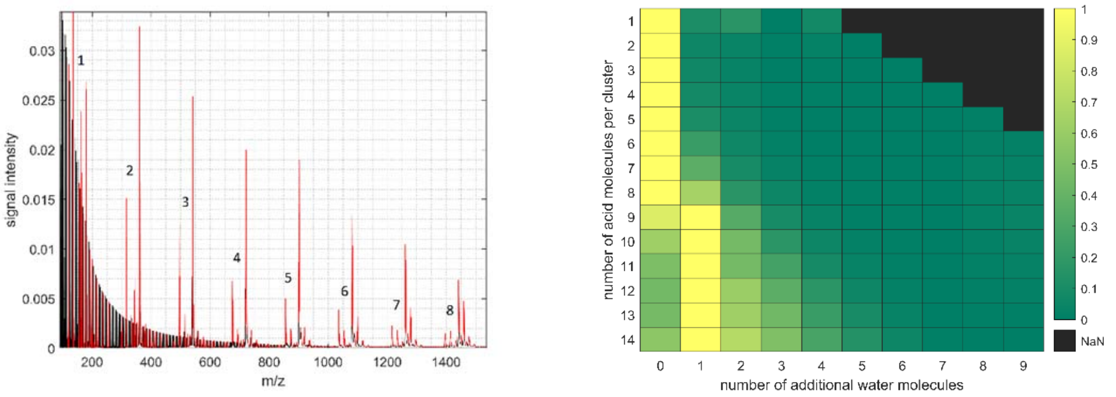

1a

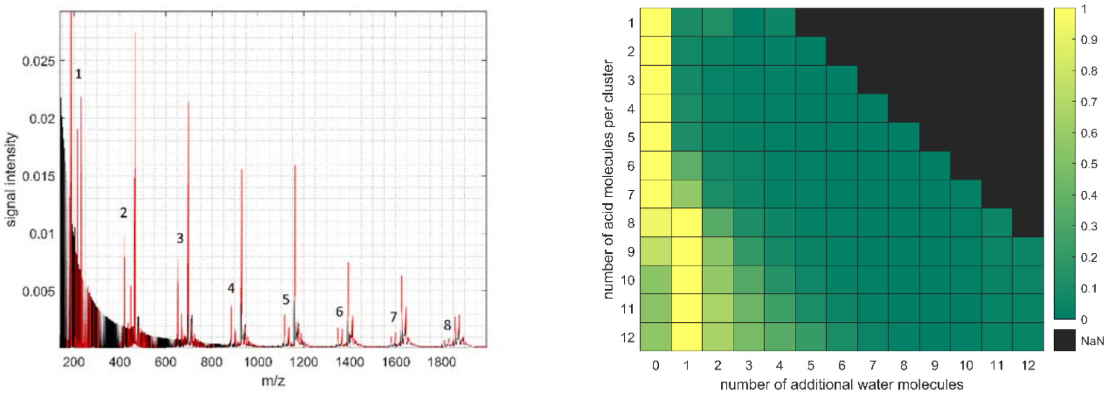

2a

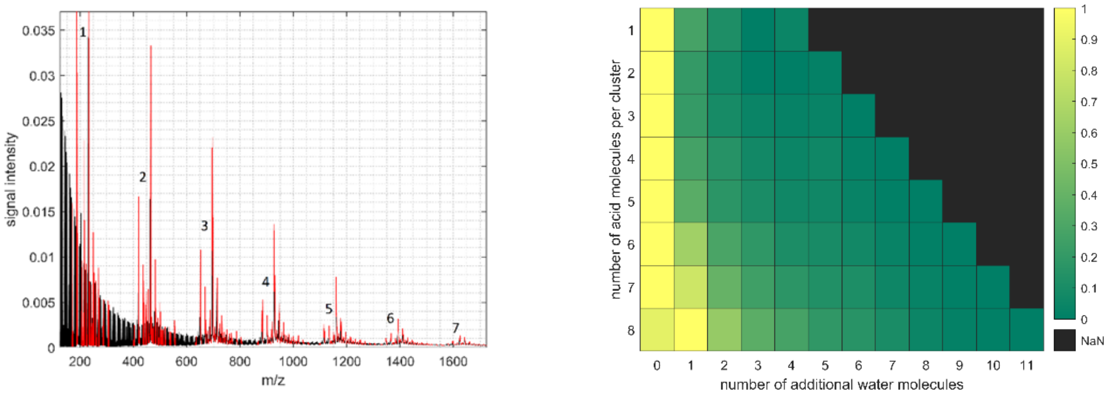

3a

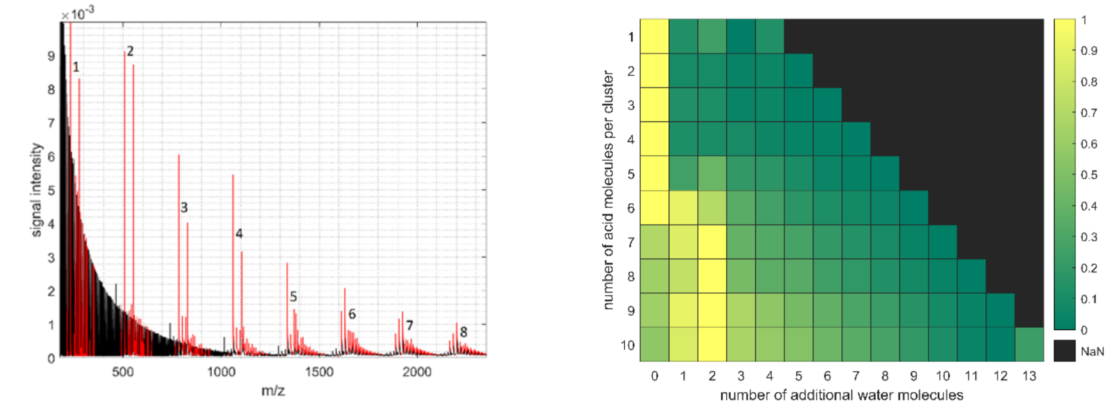

4a

Figure S1. Excerpt of the measured TOF spectrum with indication of the corresponding cluster size (left column) and integrated peak area, resolved according to attached water molecules, linewise scaled (right column) for acids **1a**, **2a**, **3a** and **4a**.

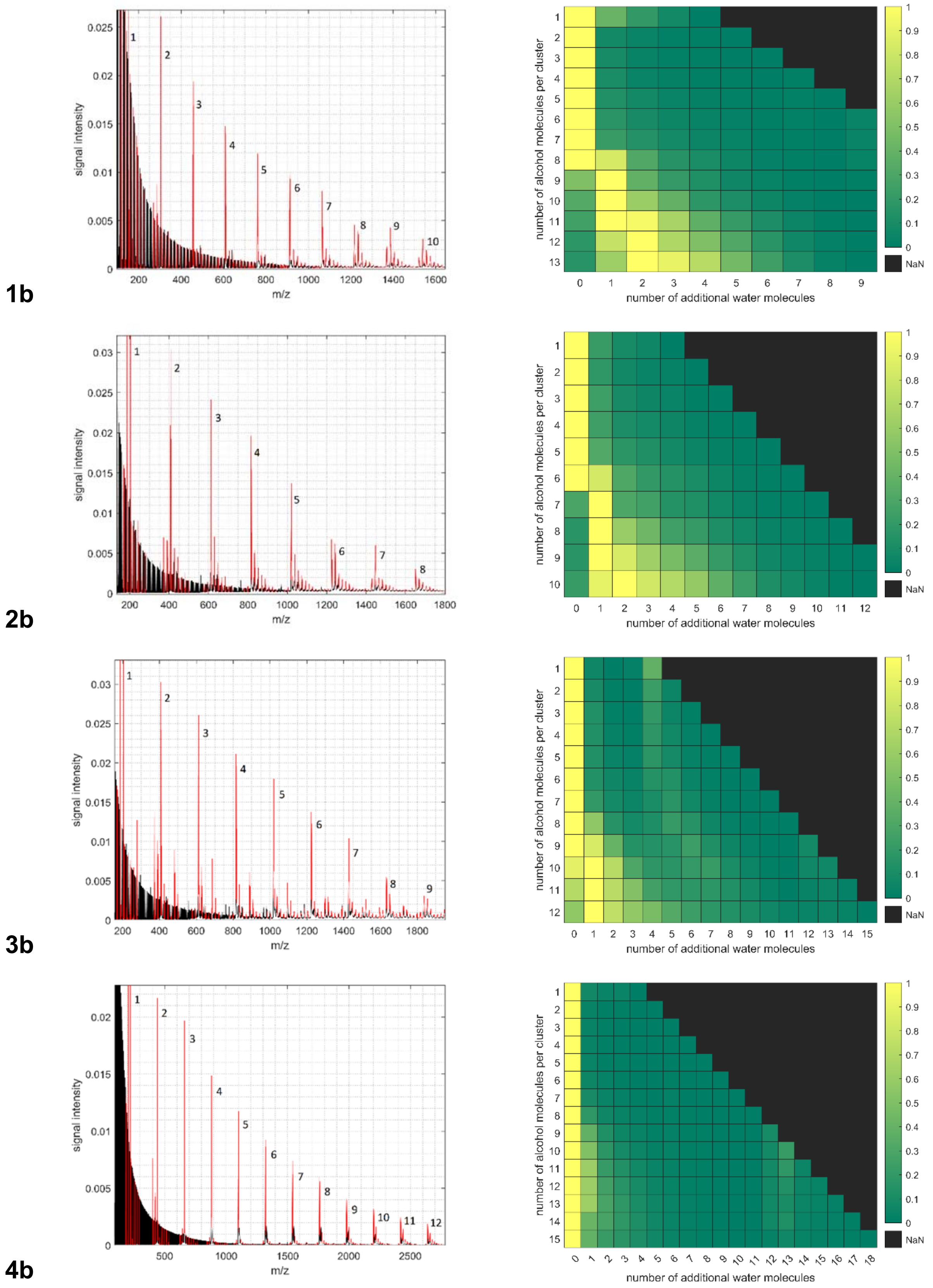

Figure S2. Excerpt of the measured TOF spectrum with indication of the corresponding cluster size (left column) and integrated peak area, resolved according to attached water molecules, linewise scaled (right column) for alcohols **1b**, **2b**, **3b** and **4b**.

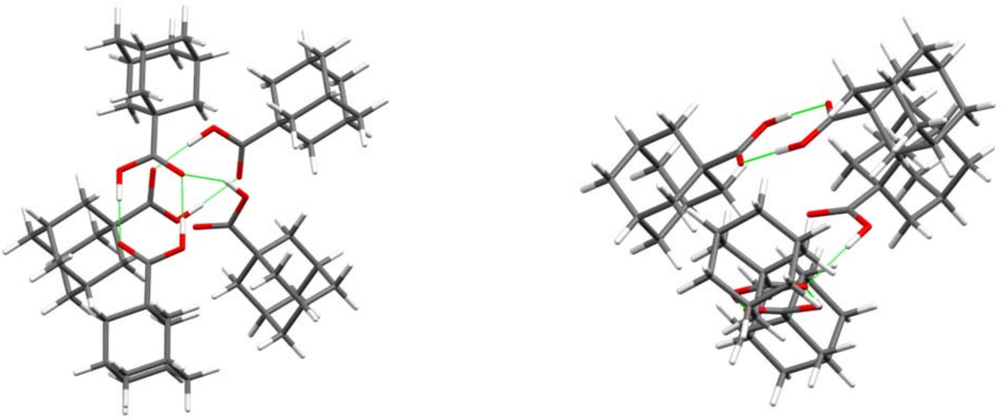

a)

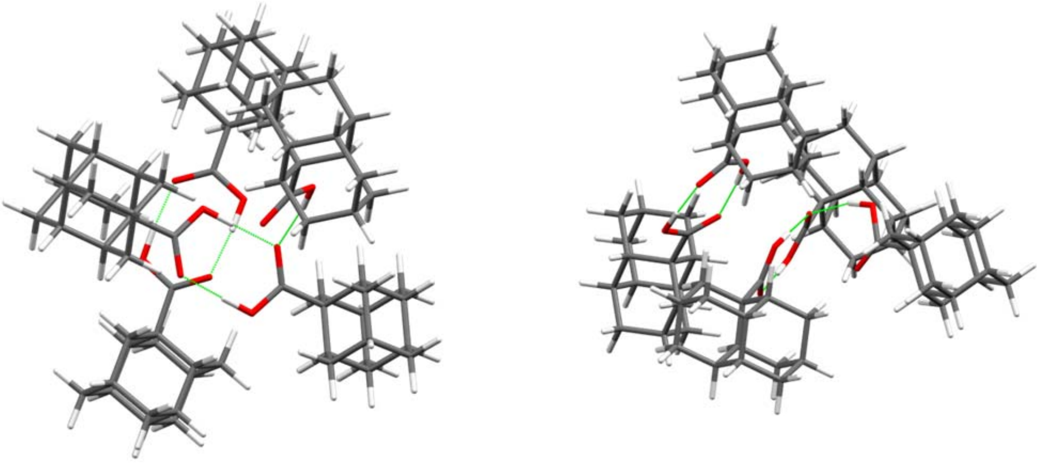

b)

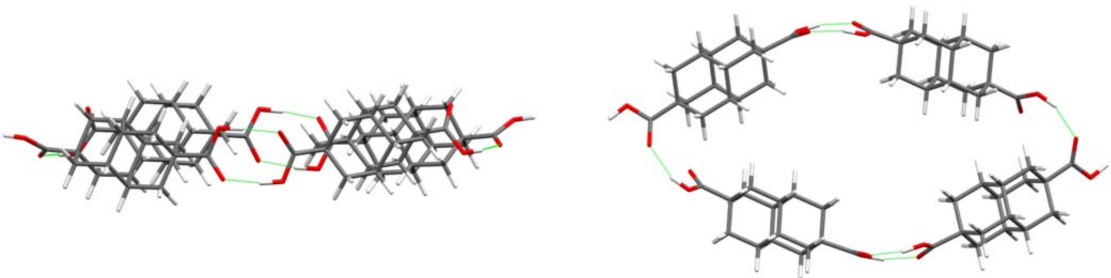

c)

Figure S3. Representations of the minimized geometries of acid clusters a) **1aCL5**, b) **2aCL5** and c) **4aCL4**.

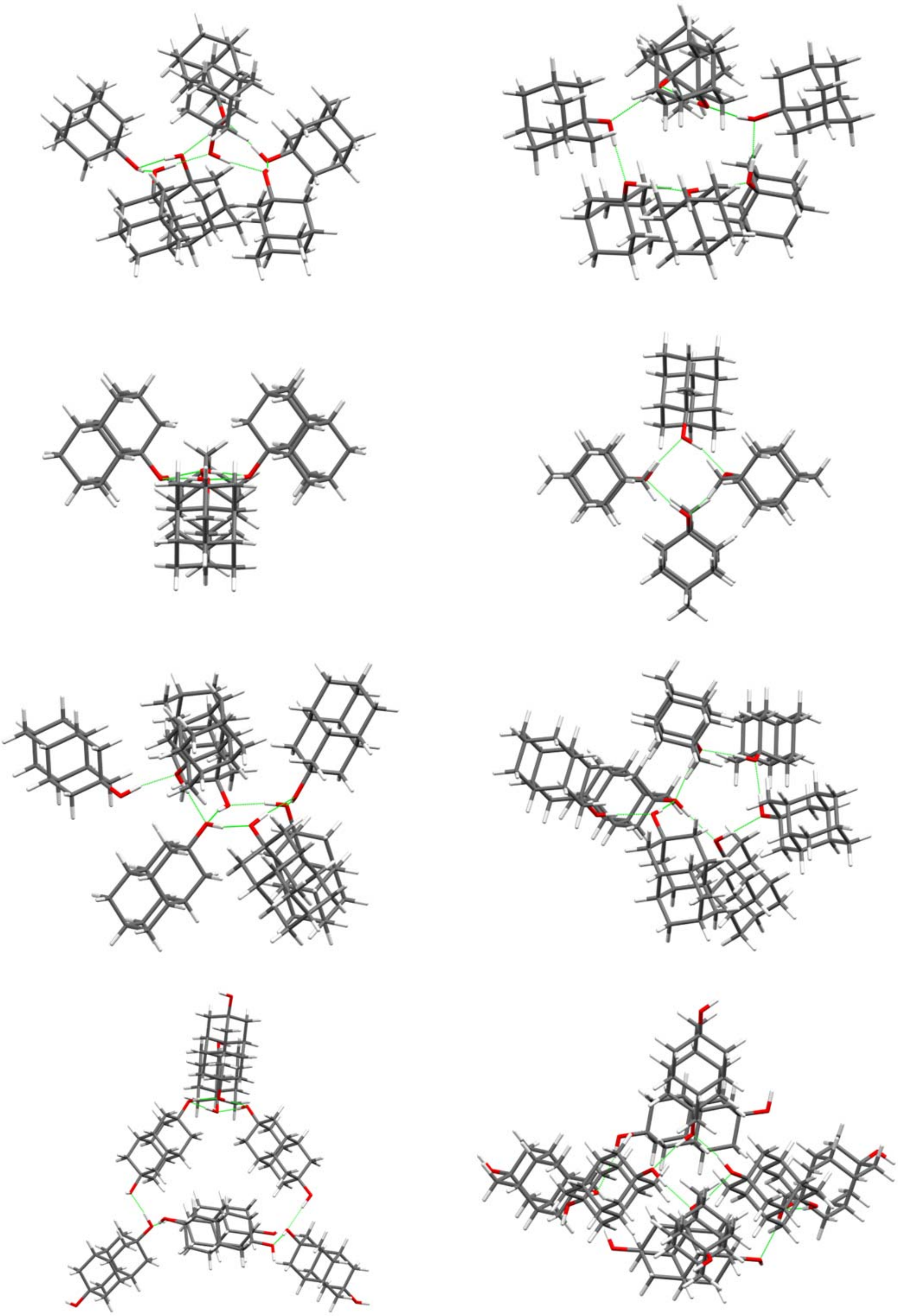

a)

b)

c)

d)

Figure S4. Representations of the minimized geometries of alcohol clusters a) **1bCL7**, b) **2bCL4**, c) **3bCL7**, and d) **4bCL8**.

Table S1. Electronic energies, zero-point vibrational energies, enthalpies and Gibbs energies of acids **1a**, **2a** and **4a** and clusters **1aCL5**, **2aCL5** and **4aCL4** in Hartree computed using the GFN2-xTB method at 0.4 K.

compound	*E*	*ZPVE*	*H*	*G*
**1a**	-39.929926	0.253488	-39.676433	-39.676444
**2a**	-50.584295	0.325164	-50.259126	-50.259139
**4a**	-60.875108	0.337936	-60.537167	-60.537178
**1aCL5**	-199.752051	1.271355	-198.480691	-198.480689
**2aCL5**	-253.024316	1.628842	-251.395470	-251.395468
**4aCL4**	-243.585872	1.355109	-242.230759	-242.230761

Table S2. Electronic energies, zero-point vibrational energies, enthalpies and Gibbs energies of alcohols **1b–4b** and clusters **1bCL7**, **2bCL4**, **3bCL7**, and **4bCL8** in Hartree computed using the GFN2-xTB method at 0.4 K.

compound	*E*	*ZPVE*	*H*	*G*
**1b**	-33.704610	0.243272	-33.461333	-33.461344
**2b**	-44.357360	0.314866	-44.042488	-44.042501
**3b**	-44.358251	0.314828	-44.043418	-44.043431
**4b**	-48.423368	0.317556	-48.105806	-48.105819
**1bCL7**	-236.047323	1.712012	-234.335306	-234.335295
**2bCL4**	-177.490809	1.264303	-176.226501	-176.226505
**3bCL7**	-310.626979	2.212890	-308.414084	-308.414073
**4bCL8**	-387.528230	2.551637	-384.976588	-384.976574

Table S3. Electronic energies of acids **1a**, **2a** and **4a** and clusters **1aCL5**, **2aCL5** and **4aCL4** in Hartree and interaction energies, Δ*H*(0 K),[a] for clusters **1aCL5**, **2aCL5** and **4aCL4** in kcal mol^{-1} computed at the B3LYP-gCP-D3(BJ)-ABC/def2-TZVPP level of theory.

compound	*E*	compound	*E*	Δ*H*
**1a**	-579.201550	**1aCL5**	-2896.093007	-51.0
**2a**	-734.016107	**2aCL5**	-3670.174230	-56.9

**4a**	-922.595452	**4aCL4**	-3690.461279	-47.8

[a] Interaction energies are defined as a difference between the energy of the cluster and the energy of the corresponding number of acid molecules. *ZPVE* taken from GFN2-xTB computations.

Table S4. Electronic energies of alcohols **1b–4b** and clusters **1bCL7**, **2bCL4**, **3bCL7**, and **4bCL8** in Hartree and interaction energies, ΔH(0 K),[a] for clusters **1bCL7**, **2bCL4**, **3bCL7**, and **4bCL8** in kcal mol^{-1} computed at the B3LYP-gCP-D3(BJ)-ABC/def2-TZVPP level of theory.

compound	*E*	compound	*E*	ΔH
**1b**	-465.850011	**1bCL7**	-3261.070347	-69.8
**2b**	-620.665476	**2bCL4**	-2482.727192	-37.9
**3b**	-620.667380	**3bCL7**	-4344.790221	-68.7
**4b**	-695.895351	**4bCL8**	-5567.294909	-75.9

[a] Interaction energies are defined as a difference between the energy of the cluster and the energy of the corresponding number of alcohol molecules. *ZPVE* taken from GFN2-xTB computations.

Table S5. Geometries of acids **1a**, **2a** and **4a** and clusters **1aCL5**, **2aCL5** and **4aCL4** in Cartesian coordinates in Å computed using the GFN2-xTB method at 0.4 K.

**1a**			
1	-0.098928000	3.524137000	-1.170693000
8	0.048681000	2.570037000	-1.105971000
6	1.152588000	0.740716000	-2.180670000
6	1.930254000	0.352272000	-0.897597000
6	2.262983000	-1.147380000	-0.928116000
1	1.330187000	0.590163000	-0.017329000
1	2.849614000	0.942277000	-0.835284000
6	2.012405000	0.409898000	-3.413153000
6	2.341650000	-1.089644000	-3.435639000
1	2.929572000	1.002464000	-3.389631000
1	1.477757000	0.699564000	-4.320802000
6	3.120445000	-1.461752000	-2.163968000
1	4.059198000	-0.900683000	-2.122692000
1	3.381848000	-2.524268000	-2.178594000
6	-0.155641000	-0.086934000	-2.234709000
6	0.958653000	-1.959145000	-0.989609000
1	2.815257000	-1.405497000	-0.020155000
6	1.038644000	-1.902115000	-3.496358000
1	2.951531000	-1.307001000	-4.316731000
6	0.180537000	-1.585998000	-2.261583000
1	-0.725934000	0.186703000	-3.127611000
1	-0.776699000	0.147052000	-1.368180000
1	0.349332000	-1.760983000	-0.102360000
1	1.185019000	-3.029735000	-0.990794000
1	-0.751471000	-2.156683000	-2.303167000
1	0.486523000	-1.661016000	-4.410145000
1	1.263371000	-2.972568000	-3.531012000
6	0.833236000	2.222616000	-2.158959000
8	1.211163000	3.039852000	-2.959584000

**2a**			
1	-2.103547000	-0.423627000	-0.570172000
6	-2.126946000	1.631517000	-3.046781000
6	-2.104412000	2.982008000	-2.281564000
6	-2.586102000	4.114006000	-3.193324000
6	-1.661950000	4.205185000	-4.413627000
6	-1.696053000	2.880072000	-5.181612000
1	-0.638751000	4.427044000	-4.094485000
1	-1.979174000	5.024188000	-5.066917000
6	-1.210211000	1.713875000	-4.294050000
1	-1.086287000	3.180483000	-1.934454000
1	-2.743667000	2.907244000	-1.398120000
6	-3.130406000	2.575697000	-5.662738000
1	-1.034294000	2.946817000	-6.051499000
6	-4.019097000	3.815047000	-3.651343000
1	-2.561704000	5.055078000	-2.636578000
6	-3.569506000	1.324679000	-3.531351000
6	-4.044785000	2.492001000	-4.423560000
1	-5.068818000	2.278686000	-4.747983000
1	-4.686838000	3.757533000	-2.785762000
1	-4.388906000	4.625711000	-4.287095000

6	-1.601133000	0.598477000	-2.064811000
6	-1.250737000	0.401866000	-5.091059000
1	-0.187715000	1.908411000	-3.963538000
6	-3.169452000	1.259960000	-6.447895000
1	-3.471759000	3.395934000	-6.303468000
6	-3.602525000	0.010059000	-4.323277000
1	-4.226997000	1.248065000	-2.663261000
6	-2.686193000	0.112354000	-5.551239000
1	-2.534548000	1.335990000	-7.336441000
1	-4.188170000	1.064534000	-6.798392000
1	-4.630177000	-0.198662000	-4.636608000
1	-3.293211000	-0.823249000	-3.685740000
1	-0.867329000	-0.419858000	-4.483498000
1	-0.590131000	0.489463000	-5.959188000
1	-2.712187000	-0.828979000	-6.107202000
8	-2.547242000	0.177394000	-1.185498000
8	-0.461995000	0.212625000	-1.989167000

**4a**			
1	0.833661000	2.250137000	-2.675955000
6	1.659620000	3.354243000	0.227105000
6	2.098272000	4.751452000	0.692025000
6	2.379931000	4.740182000	2.197098000
6	1.117586000	4.335703000	2.983203000
6	0.677484000	2.936971000	2.507964000
1	0.317454000	5.052833000	2.772043000
6	1.392054000	4.328339000	4.490193000
6	0.387275000	2.941624000	1.004058000
1	1.320286000	5.480113000	0.453320000
1	2.990017000	5.057679000	0.140518000
6	1.800240000	1.933467000	2.839070000
1	-0.232175000	2.645404000	3.043413000
6	3.501919000	3.738384000	2.533696000
1	2.690945000	5.742499000	2.508945000
6	2.783841000	2.342038000	0.549361000
6	3.063643000	2.340399000	2.055447000
1	3.863171000	1.622652000	2.266826000
1	4.412123000	4.029565000	1.999473000
6	3.788267000	3.728606000	4.037732000
6	1.375472000	3.367097000	-1.261858000
1	0.056678000	1.953050000	0.679389000
1	-0.421265000	3.644712000	0.781921000
1	1.488622000	0.931451000	2.526018000
6	2.076850000	1.918242000	4.345586000
1	2.492616000	1.344000000	0.216369000
1	3.690357000	2.615458000	0.000833000
8	0.995867000	2.148786000	-1.727385000
6	2.523786000	3.324032000	4.810932000
1	2.859288000	1.188955000	4.576895000
1	1.180666000	1.616496000	4.890974000
1	4.124123000	4.713971000	4.368601000
1	4.597394000	3.031383000	4.266603000
1	0.488237000	4.054554000	5.037821000
1	1.683160000	5.329035000	4.823862000
8	1.457530000	4.323191000	-1.989862000

1	1.986165000	2.980835000	7.960604000
8	1.716957000	2.961611000	7.031321000
6	2.806511000	3.312644000	6.299947000
8	3.859312000	3.576652000	6.822276000

**1aCL5**			
1	-1.097375000	0.700630000	-2.602029000
8	-0.217076000	0.311316000	-2.889137000
6	1.258501000	-1.551867000	-2.853802000
6	1.382750000	-1.600497000	-4.384103000
6	2.726486000	-2.224630000	-4.758600000
1	1.309158000	-0.589486000	-4.789589000
1	0.566286000	-2.195493000	-4.801545000
6	1.344888000	-2.971021000	-2.290457000
6	2.688246000	-3.587321000	-2.670193000
1	0.524932000	-3.573454000	-2.687563000
1	1.238442000	-2.940223000	-1.206106000
6	2.813064000	-3.641369000	-4.192473000
1	2.013923000	-4.258650000	-4.609594000
1	3.766822000	-4.094299000	-4.473258000
6	2.398473000	-0.698612000	-2.284704000
6	3.862775000	-1.379187000	-4.185894000
1	2.811272000	-2.260150000	-5.851018000
6	3.817619000	-2.739541000	-2.087407000
1	2.746575000	-4.602164000	-2.262207000
6	3.736312000	-1.327226000	-2.664093000
1	2.293396000	-0.644589000	-1.201102000
1	2.325558000	0.315131000	-2.682689000
1	3.818781000	-0.368092000	-4.597634000
1	4.825305000	-1.813975000	-4.465875000
1	4.550476000	-0.716445000	-2.255300000
1	3.723020000	-2.701402000	-1.000503000
1	4.784492000	-3.187318000	-2.330526000
6	-0.068779000	-0.923407000	-2.486726000
8	-0.945193000	-1.539250000	-1.899487000
1	-2.336350000	-0.996493000	-1.375432000
8	-3.209891000	-0.615255000	-1.016766000
6	-4.631862000	1.257659000	-0.766744000
6	-4.818017000	2.688826000	-1.271126000
6	-6.082703000	3.283557000	-0.656089000
1	-4.890026000	2.686389000	-2.360857000
1	-3.950390000	3.291302000	-0.998687000
6	-4.507309000	1.259462000	0.764842000
6	-5.769595000	1.866752000	1.372258000
1	-3.627973000	1.833172000	1.064494000
1	-4.378746000	0.235538000	1.119488000
6	-5.949432000	3.297063000	0.866172000
1	-5.090370000	3.905326000	1.157987000
1	-6.841861000	3.740001000	1.313983000
6	-5.847180000	0.409651000	-1.165539000
6	-7.293635000	2.442596000	-1.057547000
1	-6.212600000	4.308518000	-1.020744000
6	-6.980444000	1.024790000	0.972568000
1	-5.672337000	1.873509000	2.463615000
6	-7.108240000	1.013385000	-0.550037000

1	-5.709935000	-0.614676000	-0.814441000
1	-5.937584000	0.386525000	-2.254426000
1	-7.401195000	2.440979000	-2.144604000
1	-8.203071000	2.872636000	-0.631913000
1	-7.973256000	0.406396000	-0.839982000
1	-6.862740000	0.003994000	1.343341000
1	-7.886809000	1.440848000	1.417990000
6	-3.370261000	0.640807000	-1.339447000
8	-2.570054000	1.257542000	-2.023118000
1	1.712477000	-2.687653000	1.276753000
8	0.832599000	-3.190400000	1.258285000
6	-1.498608000	-3.208320000	1.698563000
6	-1.683519000	-3.931700000	0.360904000
6	-3.029452000	-4.652864000	0.362880000
1	-0.874054000	-4.649908000	0.220793000
1	-1.639073000	-3.211393000	-0.455976000
6	-2.633347000	-2.205338000	1.915523000
6	-3.970207000	-2.942222000	1.913000000
1	-2.620412000	-1.466531000	1.113567000
1	-2.491197000	-1.685912000	2.866266000
6	-4.156505000	-3.641734000	0.567748000
1	-4.138605000	-2.902416000	-0.234716000
1	-5.123038000	-4.150608000	0.542828000
6	-1.514889000	-4.245056000	2.835919000
6	-3.051613000	-5.684667000	1.490271000
1	-3.164567000	-5.159907000	-0.598437000
6	-3.989906000	-3.971675000	3.041681000
1	-4.780273000	-2.219723000	2.066416000
6	-2.857460000	-4.975085000	2.829467000
1	-1.366828000	-3.743251000	3.795811000
1	-0.698668000	-4.956390000	2.693132000
1	-2.256549000	-6.418187000	1.338972000
1	-4.004557000	-6.218741000	1.490245000
1	-2.864354000	-5.712475000	3.640351000
1	-3.869371000	-3.470616000	4.005178000
1	-4.949383000	-4.493577000	3.053711000
6	-0.158232000	-2.506284000	1.746455000
8	-0.019491000	-1.396008000	2.250227000
1	1.413925000	-0.549256000	2.345049000
8	2.290852000	-0.057977000	2.377264000
6	4.556151000	0.038884000	1.681616000
6	4.362050000	1.194565000	0.688194000
6	5.660858000	1.987869000	0.571545000
1	3.556490000	1.840061000	1.042452000
1	4.074197000	0.795925000	-0.286687000
6	5.675074000	-0.875856000	1.181753000
6	6.970479000	-0.075264000	1.066183000
1	5.400719000	-1.293758000	0.211957000
1	5.805464000	-1.709044000	1.875628000
6	6.777008000	1.070901000	0.074194000
1	6.520054000	0.672004000	-0.909724000
1	7.705659000	1.637083000	-0.026250000
6	4.931097000	0.613482000	3.052884000
6	6.034531000	2.557391000	1.939188000
1	5.515815000	2.808268000	-0.140756000

6	7.347292000	0.491955000	2.434349000
1	7.770093000	-0.734483000	0.710991000
6	6.229465000	1.409211000	2.928625000
1	4.126544000	1.256119000	3.414516000
1	5.060458000	-0.201684000	3.769099000
1	5.244929000	3.224109000	2.293827000
1	6.954732000	3.140499000	1.860560000
1	6.495985000	1.814906000	3.910994000
1	8.281778000	1.052622000	2.360050000
1	7.501623000	-0.322941000	3.145186000
6	3.242432000	-0.714213000	1.766541000
8	3.088448000	-1.829231000	1.296747000
1	-0.845141000	0.182597000	1.858742000
8	-0.875099000	1.125827000	1.580469000
6	0.198658000	2.790742000	0.259898000
6	1.105075000	2.930401000	-0.963692000
6	1.346745000	4.409505000	-1.251765000
1	0.633948000	2.449082000	-1.822003000
1	2.050995000	2.419757000	-0.777513000
6	0.862368000	3.454162000	1.477256000
6	1.111137000	4.930432000	1.175940000
1	1.804745000	2.950649000	1.704827000
1	0.210689000	3.350793000	2.347160000
6	2.020390000	5.061945000	-0.045007000
1	2.980294000	4.577907000	0.151157000
1	2.213714000	6.117356000	-0.250786000
6	-1.139198000	3.484460000	-0.012787000
6	0.011115000	5.097206000	-1.530857000
1	1.997204000	4.505345000	-2.128266000
6	-0.222305000	5.622890000	0.898187000
1	1.592137000	5.397166000	2.043514000
6	-0.892463000	4.963239000	-0.306280000
1	-1.788660000	3.378153000	0.858753000
1	-1.623115000	3.011558000	-0.868814000
1	-0.468581000	4.635500000	-2.396268000
1	0.174749000	6.153194000	-1.758559000
1	-1.852013000	5.454871000	-0.504734000
1	-0.870297000	5.546089000	1.774498000
1	-0.055730000	6.683613000	0.696701000
6	-0.003910000	1.325931000	0.596631000
8	0.586653000	0.415582000	0.066063000

**2aCL5**			
1	1.415783000	-0.931005000	-1.414410000
6	1.318824000	-3.448210000	0.699842000
6	2.112654000	-3.017494000	1.947770000
6	2.505326000	-4.248551000	2.756257000
6	1.242694000	-4.987471000	3.193447000
6	0.454720000	-5.417957000	1.957920000
1	0.627935000	-4.335283000	3.817971000
1	1.509974000	-5.865819000	3.785652000
6	0.048569000	-4.197659000	1.128075000
1	1.496507000	-2.346198000	2.550081000
1	3.002284000	-2.469012000	1.634473000
6	1.306926000	-6.346924000	1.090100000

1	-0.451649000	-5.948106000	2.273114000
6	3.366544000	-5.165211000	1.891101000
1	3.072915000	-3.933238000	3.638645000
6	2.167405000	-4.376796000	-0.175072000
6	2.570624000	-5.596016000	0.660916000
1	3.190000000	-6.257466000	0.043874000
1	4.272791000	-4.639170000	1.581857000
1	3.667984000	-6.045207000	2.464248000
6	0.965353000	-2.142465000	0.018679000
6	-0.738194000	-4.637861000	-0.106529000
1	-0.571052000	-3.533103000	1.740105000
6	0.512160000	-6.796217000	-0.134027000
1	1.593800000	-7.226680000	1.678478000
6	1.380458000	-4.836543000	-1.403181000
1	3.066764000	-3.842728000	-0.498857000
6	0.118801000	-5.572333000	-0.958011000
1	-0.382480000	-7.337442000	0.183448000
1	1.116274000	-7.474730000	-0.740989000
1	2.005124000	-5.501131000	-2.004871000
1	1.115159000	-3.979326000	-2.022687000
1	-1.027214000	-3.765236000	-0.697650000
1	-1.651157000	-5.150757000	0.206732000
1	-0.451118000	-5.888988000	-1.838556000
8	1.657313000	-1.841950000	-1.033212000
8	0.109862000	-1.399229000	0.493094000
1	1.603693000	0.427546000	1.845325000
6	-0.314386000	2.271648000	3.746330000
6	0.520023000	2.332402000	5.040350000
6	-0.379540000	2.708467000	6.213448000
6	-1.007942000	4.074630000	5.949804000
6	-1.838260000	4.011167000	4.669777000
1	-0.225837000	4.830752000	5.848509000
1	-1.644120000	4.360350000	6.790802000
6	-0.957539000	3.637356000	3.475075000
1	1.315751000	3.072815000	4.924641000
1	0.982537000	1.358513000	5.218726000
6	-2.946930000	2.964971000	4.815020000
1	-2.289898000	4.992525000	4.483215000
6	-1.475814000	1.656588000	6.363948000
1	0.222084000	2.749038000	7.128369000
6	-1.423452000	1.222373000	3.887312000
6	-2.303860000	1.601495000	5.081882000
1	-3.091283000	0.846945000	5.192926000
1	-1.029115000	0.679105000	6.560678000
1	-2.119029000	1.908147000	7.210471000
6	0.687473000	1.911317000	2.666262000
6	-1.802258000	3.578350000	2.203050000
1	-0.165785000	4.383799000	3.348849000
6	-3.793276000	2.909735000	3.545048000
1	-3.585677000	3.234265000	5.664826000
6	-2.266225000	1.169985000	2.613916000
1	-0.970391000	0.241653000	4.062810000
6	-2.904034000	2.533967000	2.362420000
1	-4.260656000	3.881549000	3.368240000
1	-4.590241000	2.171333000	3.662358000

1	-3.038342000	0.405203000	2.722624000
1	-1.642005000	0.896201000	1.763586000
1	-1.168318000	3.317319000	1.354550000
1	-2.240254000	4.560966000	2.009521000
1	-3.505132000	2.494050000	1.447322000
8	0.916511000	0.628945000	2.551728000
8	1.293387000	2.753213000	2.022376000
1	-0.165477000	0.129978000	-0.022814000
6	-0.043070000	2.669690000	-2.073293000
6	0.829040000	3.650458000	-1.267964000
6	0.611059000	5.070037000	-1.779335000
6	1.006230000	5.139479000	-3.252334000
6	0.142223000	4.167759000	-4.053213000
1	2.061424000	4.880110000	-3.367289000
1	0.867676000	6.155704000	-3.629282000
6	0.340136000	2.732388000	-3.557768000
1	1.880349000	3.375073000	-1.377591000
1	0.573241000	3.590694000	-0.208433000
6	-1.335446000	4.543018000	-3.910891000
1	0.426580000	4.218476000	-5.110904000
6	-0.860395000	5.444026000	-1.620646000
1	1.231919000	5.758433000	-1.195942000
6	-1.525040000	3.041082000	-1.931936000
6	-1.719468000	4.475164000	-2.430436000
1	-2.775753000	4.747885000	-2.318390000
1	-1.142107000	5.398971000	-0.566236000
1	-1.025392000	6.466022000	-1.970336000
6	0.241869000	1.301357000	-1.488586000
6	-0.528132000	1.777496000	-4.377185000
1	1.391840000	2.445458000	-3.665858000
6	-2.205317000	3.588442000	-4.725924000
1	-1.482864000	5.567347000	-4.274238000
6	-2.385638000	2.077929000	-2.749158000
1	-1.812810000	2.978675000	-0.877451000
6	-1.997179000	2.161600000	-4.223290000
1	-1.937897000	3.654142000	-5.783398000
1	-3.256782000	3.870562000	-4.628426000
1	-3.440299000	2.342146000	-2.628454000
1	-2.244359000	1.056620000	-2.389353000
1	-0.374841000	0.752127000	-4.038949000
1	-0.235057000	1.831757000	-5.428547000
1	-2.618535000	1.471327000	-4.804703000
8	-0.356546000	1.065066000	-0.351333000
8	1.027347000	0.515536000	-1.995209000
1	2.615158000	2.552981000	1.061018000
6	4.672884000	0.733868000	-0.712969000
6	4.179250000	1.187142000	-2.099943000
6	5.240788000	0.881235000	-3.149924000
6	6.519289000	1.641266000	-2.805851000
6	7.013356000	1.198550000	-1.430527000
1	6.324647000	2.716441000	-2.803296000
1	7.287770000	1.441342000	-3.556303000
6	5.961062000	1.490511000	-0.356445000
1	3.983003000	2.262019000	-2.072026000
1	3.247582000	0.675365000	-2.345926000

6	7.309732000	-0.303140000	-1.442746000
1	7.930522000	1.745731000	-1.181971000
6	5.516082000	-0.619992000	-3.165785000
1	4.874770000	1.199315000	-4.132204000
6	4.971161000	-0.770538000	-0.718216000
6	6.022908000	-1.056091000	-1.793143000
1	6.228789000	-2.132853000	-1.809479000
1	4.599557000	-1.160920000	-3.410333000
1	6.262269000	-0.854900000	-3.928560000
6	3.544577000	1.071670000	0.240973000
6	6.476075000	1.042814000	1.011358000
1	5.749696000	2.564639000	-0.331822000
6	7.832071000	-0.745641000	-0.077849000
1	8.068611000	-0.513388000	-2.205820000
6	5.497205000	-1.218635000	0.646152000
1	4.049589000	-1.313693000	-0.949793000
6	6.774875000	-0.455037000	0.984570000
1	8.755747000	-0.211188000	0.157284000
1	8.058632000	-1.814477000	-0.094746000
1	5.701214000	-2.292401000	0.622360000
1	4.744522000	-1.036718000	1.414826000
1	5.730138000	1.260737000	1.779862000
1	7.382841000	1.599431000	1.260417000
1	7.142906000	-0.772631000	1.966901000
8	3.416345000	2.351925000	0.482245000
8	2.784033000	0.236593000	0.704901000
1	-1.605784000	-1.583792000	0.776272000
6	-4.387348000	-1.251066000	-0.848381000
6	-4.811535000	0.107709000	-0.263377000
6	-6.307776000	0.314546000	-0.472555000
6	-6.620059000	0.279504000	-1.966485000
6	-6.206966000	-1.074641000	-2.538803000
1	-6.078334000	1.078177000	-2.478762000
1	-7.688616000	0.440836000	-2.128275000
6	-4.705824000	-1.303159000	-2.347706000
1	-4.247321000	0.905333000	-0.751219000
1	-4.575547000	0.127774000	0.802370000
6	-6.972389000	-2.198906000	-1.835473000
1	-6.435744000	-1.098421000	-3.610742000
6	-7.071422000	-0.799323000	0.239595000
1	-6.598953000	1.285813000	-0.056505000
6	-5.151967000	-2.378145000	-0.142532000
6	-6.653343000	-2.149430000	-0.339179000
1	-7.202871000	-2.948014000	0.173412000
1	-6.856633000	-0.771146000	1.310536000
1	-8.146923000	-0.657657000	0.108954000
6	-2.891308000	-1.336078000	-0.602693000
6	-4.309211000	-2.658978000	-2.931457000
1	-4.142684000	-0.515262000	-2.858104000
6	-6.574112000	-3.555148000	-2.413753000
1	-8.048963000	-2.046394000	-1.979177000
6	-4.748605000	-3.732092000	-0.725023000
1	-4.914430000	-2.356717000	0.926019000
6	-5.074905000	-3.769277000	-2.216486000
1	-6.818451000	-3.590910000	-3.478139000

1	-7.134511000	-4.349612000	-1.914552000
1	-5.285884000	-4.529513000	-0.205264000
1	-3.679526000	-3.894730000	-0.574756000
1	-3.234292000	-2.806955000	-2.820000000
1	-4.538950000	-2.676141000	-3.999598000
1	-4.784598000	-4.741487000	-2.630659000
8	-2.584514000	-1.620159000	0.656160000
8	-2.047720000	-1.108096000	-1.437742000

**4aCL4**			
1	8.756752000	-2.659283000	-0.412816000
6	5.699476000	-3.337136000	0.426099000
6	5.099642000	-2.741969000	-0.854892000
6	3.656271000	-3.212733000	-1.007085000
6	3.602643000	-4.738812000	-1.103684000
6	4.216673000	-5.331879000	0.167067000
1	4.183953000	-5.065654000	-1.973119000
6	2.157225000	-5.206602000	-1.269093000
6	5.664555000	-4.866285000	0.324231000
1	5.692690000	-3.056150000	-1.715523000
1	5.126005000	-1.650530000	-0.804761000
6	3.379837000	-4.888143000	1.370291000
1	4.201826000	-6.425656000	0.099622000
6	2.827406000	-2.763609000	0.197986000
1	3.233688000	-2.777204000	-1.919965000
6	4.874049000	-2.891761000	1.633638000
6	3.432621000	-3.361852000	1.469660000
1	2.847414000	-3.035546000	2.336783000
1	2.848261000	-1.669381000	0.266006000
6	1.381283000	-3.220250000	0.037948000
6	7.131179000	-2.846595000	0.572987000
1	6.100006000	-5.304002000	1.225558000
1	6.259948000	-5.188635000	-0.532041000
1	3.799847000	-5.323471000	2.283770000
6	1.934607000	-5.357266000	1.214787000
1	5.309031000	-3.306007000	2.544854000
1	4.908832000	-1.803525000	1.721773000
8	7.887570000	-3.117628000	-0.483145000
6	1.338714000	-4.756497000	-0.058082000
1	1.340734000	-5.050717000	2.077691000
1	1.896762000	-6.447242000	1.157258000
1	0.948038000	-2.786807000	-0.866460000
1	0.787817000	-2.890395000	0.894446000
1	2.124664000	-6.295521000	-1.349129000
1	1.729274000	-4.788667000	-2.181500000
8	7.539353000	-2.237005000	1.531578000
1	-1.603367000	-5.003838000	-1.436919000
8	-0.602781000	-4.929354000	-1.406381000
6	-0.132235000	-5.096570000	-0.194751000
8	-0.828625000	-5.416657000	0.752401000
1	2.284450000	4.825804000	-1.492090000
6	5.105984000	3.632658000	-0.364979000
6	5.805186000	3.587440000	-1.723346000
6	7.024440000	2.670758000	-1.633500000
6	8.009353000	3.185358000	-0.580340000

6	7.294144000	3.246112000	0.772296000
1	8.343143000	4.191550000	-0.858489000
6	9.230471000	2.267095000	-0.497827000
6	6.075784000	4.164135000	0.691103000
1	6.114679000	4.594839000	-2.010177000
1	5.116130000	3.218922000	-2.484574000
6	6.864569000	1.830009000	1.160793000
1	7.983561000	3.635609000	1.529498000
6	6.591469000	1.254687000	-1.247880000
1	7.520336000	2.642749000	-2.610455000
6	4.662386000	2.210425000	0.026916000
6	5.886663000	1.305129000	0.108934000
1	5.567396000	0.295617000	0.392646000
1	5.896801000	0.870366000	-2.003938000
6	7.803113000	0.332449000	-1.171945000
6	3.822507000	4.440013000	-0.393738000
1	5.572304000	4.213974000	1.658061000
1	6.386368000	5.177011000	0.425037000
1	6.366877000	1.855631000	2.136394000
6	8.079673000	0.912416000	1.254768000
1	4.151719000	2.240765000	0.992511000
1	3.961792000	1.827881000	-0.719695000
8	3.198962000	4.411225000	-1.544690000
6	8.778733000	0.853904000	-0.115861000
1	7.774298000	-0.090232000	1.558489000
1	8.779167000	1.295554000	2.000601000
1	8.302851000	0.285287000	-2.142348000
1	7.482484000	-0.675570000	-0.914620000
1	9.932016000	2.646184000	0.246620000
1	9.741168000	2.243480000	-1.463638000
8	3.374073000	4.994433000	0.594571000
1	11.687408000	-0.214906000	0.787695000
8	10.990080000	0.447489000	0.654931000
6	9.941809000	-0.098472000	0.041511000
8	9.948457000	-1.257841000	-0.304404000
1	1.766647000	5.268883000	0.937247000
6	-1.330813000	4.772376000	0.041393000
6	-1.983034000	5.441675000	1.251459000
6	-3.433209000	4.972531000	1.367182000
6	-4.218487000	5.340208000	0.105015000
6	-3.546866000	4.678527000	-1.101156000
1	-4.205058000	6.428259000	-0.025659000
6	-5.669996000	4.875011000	0.226498000
6	-2.098550000	5.146113000	-1.226760000
1	-1.950198000	6.526971000	1.132038000
1	-1.432690000	5.186098000	2.158099000
6	-3.598079000	3.160441000	-0.918192000
1	-4.090427000	4.950621000	-2.012712000
6	-3.484154000	3.454154000	1.553017000
1	-3.894991000	5.457153000	2.234635000
6	-1.371043000	3.243938000	0.225794000
6	-2.821097000	2.787409000	0.345987000
1	-2.840371000	1.698880000	0.476527000
1	-2.936158000	3.182511000	2.462278000
6	-4.929375000	2.984349000	1.681164000

6	0.142102000	5.105803000	-0.089100000
1	-1.622854000	4.682650000	-2.092912000
1	-2.064227000	6.228372000	-1.369275000
1	-3.134320000	2.675483000	-1.785020000
6	-5.044760000	2.689947000	-0.801658000
1	-0.895122000	2.759547000	-0.630707000
1	-0.815971000	2.970648000	1.126167000
8	0.771484000	5.201557000	1.055582000
6	-5.702987000	3.354681000	0.415585000
1	-5.068993000	1.603179000	-0.689060000
1	-5.600852000	2.950268000	-1.704063000
1	-5.405178000	3.447191000	2.547581000
1	-4.963776000	1.902818000	1.829684000
1	-6.228935000	5.143572000	-0.671723000
1	-6.145923000	5.361459000	1.081196000
8	0.703264000	5.192560000	-1.167668000
1	-8.726707000	2.649124000	-0.516212000
8	-7.859267000	3.114432000	-0.559850000
6	-7.134950000	2.854457000	0.521072000
8	-7.566831000	2.242440000	1.467729000
1	-11.730287000	0.221784000	0.544142000
6	-8.778027000	-0.858131000	-0.189320000
6	-9.213446000	-2.272182000	-0.586456000
6	-7.992587000	-3.194284000	-0.599568000
6	-6.951433000	-2.687955000	-1.601426000
6	-6.534577000	-1.271426000	-1.200382000
1	-7.394209000	-2.663846000	-2.603567000
6	-5.734396000	-3.609580000	-1.617814000
6	-7.745672000	-0.345417000	-1.194529000
1	-9.672389000	-2.252717000	-1.577963000
1	-9.954524000	-2.644953000	0.122040000
6	-5.903414000	-1.316728000	0.192551000
1	-5.799247000	-0.893336000	-1.920237000
6	-7.350840000	-3.249891000	0.789598000
1	-8.314123000	-4.200896000	-0.890288000
6	-8.152492000	-0.909716000	1.216711000
6	-6.938099000	-1.832858000	1.193075000
1	-6.493491000	-1.854512000	2.194082000
1	-8.081329000	-3.633121000	1.510793000
6	-6.132115000	-4.172867000	0.782074000
6	-9.944721000	0.098405000	-0.099450000
1	-7.435958000	0.662720000	-0.925303000
1	-8.192404000	-0.302085000	-2.190615000
1	-5.597137000	-0.306720000	0.488340000
6	-4.680024000	-2.226765000	0.183336000
1	-7.857746000	0.093971000	1.527795000
1	-8.891940000	-1.283983000	1.927734000
8	-11.028557000	-0.443052000	0.453286000
6	-5.106467000	-3.648492000	-0.224953000
1	-4.223628000	-2.255123000	1.175129000
1	-3.937602000	-1.849316000	-0.525072000
1	-6.433283000	-5.185649000	0.504400000
1	-5.685609000	-4.213667000	1.776716000
1	-4.999451000	-3.253834000	-2.342162000
1	-6.028346000	-4.618505000	-1.914690000

8	-9.927439000	1.257641000	-0.445566000
1	-2.460890000	-5.147782000	0.949011000
8	-3.382150000	-4.748394000	0.973780000
6	-3.829872000	-4.467018000	-0.224926000
8	-3.238773000	-4.769351000	-1.246270000

Table S6. Geometries of alcohols **1b–4b** and clusters **1bCL7**, **2bCL4**, **3bCL7**, and **4bCL8** in Cartesian coordinates in Å computed using the GFN2-xTB method at 0.4 K.

**1b**			
6	-2.925751000	-1.033629000	-3.101307000
6	-2.419229000	-1.598784000	-4.430571000
6	-3.439163000	-1.307889000	-5.544216000
1	-2.258735000	-2.674712000	-4.321805000
1	-1.451090000	-1.147159000	-4.662883000
6	-3.120233000	0.483301000	-3.238014000
6	-4.145824000	0.783843000	-4.345007000
1	-2.156080000	0.945518000	-3.466056000
1	-3.462651000	0.896956000	-2.282232000
6	-3.632001000	0.211935000	-5.676815000
1	-2.685592000	0.688974000	-5.949076000
1	-4.344733000	0.431175000	-6.477662000
6	-4.270505000	-1.688762000	-2.753212000
6	-4.785004000	-1.963235000	-5.190835000
1	-3.067951000	-1.715232000	-6.488456000
6	-5.488855000	0.125113000	-3.988484000
1	-4.278202000	1.865579000	-4.434675000
6	-5.300134000	-1.395243000	-3.858299000
1	-4.629087000	-1.303278000	-1.791555000
1	-4.121728000	-2.765709000	-2.636478000
1	-4.665961000	-3.048375000	-5.115322000
1	-5.514058000	-1.774651000	-5.984926000
1	-6.253796000	-1.864733000	-3.601621000
1	-5.872446000	0.537604000	-3.049856000
1	-6.231346000	0.345941000	-4.761404000
8	-1.929753000	-1.342183000	-2.121258000
1	-2.218584000	-0.997911000	-1.270075000

**2b**			
6	2.052403000	0.347313000	1.522554000
6	2.551697000	-0.280669000	0.217447000
6	4.084295000	-0.341933000	0.200261000
6	4.644376000	1.080810000	0.330690000
6	4.157439000	1.707798000	1.641854000
1	4.322617000	1.691890000	-0.518941000
1	5.738516000	1.058292000	0.311392000
6	2.613590000	1.780070000	1.675884000
1	2.187580000	0.312940000	-0.630074000
1	2.120585000	-1.281100000	0.121263000
6	4.630734000	0.870402000	2.849042000
1	4.556793000	2.723575000	1.732838000
6	4.557584000	-1.189428000	1.389445000
1	4.424680000	-0.791817000	-0.736568000

6	2.524108000	-0.485281000	2.730202000
6	4.067400000	-0.558478000	2.697453000
1	4.402934000	-1.165724000	3.544608000
1	4.174494000	-2.210708000	1.298800000
1	5.650174000	-1.256516000	1.393285000
8	0.618425000	0.341804000	1.549967000
6	2.136314000	2.413057000	2.989599000
1	2.264674000	2.380257000	0.825919000
6	4.152108000	1.501013000	4.161920000
1	5.725088000	0.824027000	2.841364000
6	2.048548000	0.145838000	4.046078000
1	2.106006000	-1.491673000	2.629707000
6	2.618747000	1.565637000	4.176242000
1	4.573779000	2.505942000	4.268849000
1	4.512597000	0.911462000	5.011245000
1	2.383363000	-0.472318000	4.885315000
1	0.958110000	0.169427000	4.073499000
1	1.046935000	2.483575000	2.995912000
1	2.529883000	3.431606000	3.068249000
1	2.276364000	2.015775000	5.112334000
1	0.297537000	0.814471000	0.775532000

**3b**			
6	-6.998842000	-4.405294000	1.644707000
6	-7.568670000	-3.832092000	0.339294000
6	-6.916350000	-2.476308000	0.038813000
6	-7.189641000	-1.478193000	1.184134000
6	-6.630805000	-2.070959000	2.495412000
1	-8.270919000	-1.339727000	1.286785000
6	-6.535676000	-0.124349000	0.883763000
6	-7.284946000	-3.426456000	2.792951000
1	-8.653646000	-3.712905000	0.422958000
1	-7.385947000	-4.525742000	-0.487762000
6	-5.103140000	-2.229920000	2.342951000
1	-6.836683000	-1.376360000	3.316586000
6	-5.388412000	-2.634717000	-0.111422000
1	-7.327124000	-2.071771000	-0.892366000
6	-5.480459000	-4.582257000	1.498377000
6	-4.825960000	-3.227515000	1.198147000
1	-3.743438000	-3.360206000	1.094287000
1	-5.179149000	-3.324642000	-0.935607000
6	-4.739106000	-1.276469000	-0.406986000
1	-7.464290000	-5.371647000	1.858581000
1	-6.899712000	-3.829393000	3.735220000
1	-8.365053000	-3.299077000	2.917976000
1	-4.688782000	-2.629613000	3.274553000
6	-4.456457000	-0.871847000	2.041288000
1	-5.060776000	-5.003642000	2.417515000
1	-5.259778000	-5.289625000	0.692544000
6	-5.024175000	-0.297021000	0.738254000
1	-3.369716000	-0.984281000	1.948879000
1	-4.642680000	-0.164871000	2.854521000
1	-5.124589000	-0.856740000	-1.340349000
1	-3.655604000	-1.395996000	-0.525868000
1	-6.737856000	0.589935000	1.686598000

1	-6.936202000	0.303020000	-0.039641000
8	-4.487362000	1.000383000	0.463277000
1	-3.533625000	0.923259000	0.359197000

**4b**			
6	7.153208000	-0.535181000	1.470931000
6	7.810822000	0.201583000	2.644080000
6	7.433265000	-0.474943000	3.968117000
6	5.902981000	-0.460857000	4.168646000
6	5.248376000	-1.189044000	2.975891000
1	5.554382000	0.576776000	4.189995000
6	5.526415000	-1.145098000	5.487716000
6	5.630959000	-0.509545000	1.654757000
1	7.483730000	1.247844000	2.650250000
1	8.895222000	0.200145000	2.504186000
6	5.726356000	-2.656391000	2.980353000
1	4.160459000	-1.165575000	3.094767000
6	7.911245000	-1.942284000	3.972587000
1	7.907964000	0.059028000	4.797450000
6	7.633180000	-1.986244000	1.460734000
6	7.256641000	-2.670473000	2.779831000
1	7.605250000	-3.708103000	2.758480000
1	8.999160000	-1.965771000	3.853704000
6	7.528648000	-2.621790000	5.293710000
8	7.537820000	0.031084000	0.215287000
1	5.159556000	-1.015446000	0.807914000
1	5.279470000	0.528748000	1.651274000
1	5.251677000	-3.190344000	2.150997000
6	5.348773000	-3.332925000	4.304377000
1	7.181656000	-2.505806000	0.611384000
1	8.716272000	-2.002362000	1.312462000
6	6.006385000	-2.596167000	5.477525000
1	5.675849000	-4.379194000	4.298191000
1	4.264368000	-3.331454000	4.444257000
1	7.999975000	-2.115866000	6.140581000
1	7.880162000	-3.660069000	5.297232000
1	4.443319000	-1.128987000	5.635968000
1	5.977904000	-0.625572000	6.337108000
1	7.250697000	0.949437000	0.190251000
8	5.621803000	-3.162395000	6.733186000
1	5.907893000	-4.081081000	6.757789000

**1bCL7**			
6	-4.379107000	-0.986743000	0.963356000
6	-4.414829000	-2.252101000	1.823489000
6	-5.565660000	-3.147619000	1.370601000
1	-4.538647000	-1.968838000	2.870587000
1	-3.463595000	-2.779510000	1.732036000
6	-4.195436000	-1.369130000	-0.507150000
6	-5.344768000	-2.269308000	-0.954157000
1	-3.242605000	-1.886399000	-0.628201000
1	-4.163934000	-0.458641000	-1.109826000
6	-5.366986000	-3.533269000	-0.094969000
1	-4.427405000	-4.078094000	-0.211291000
1	-6.177833000	-4.189575000	-0.419374000

6	-5.707918000	-0.237092000	1.114980000
6	-6.888523000	-2.398951000	1.527878000
1	-5.582733000	-4.053263000	1.987652000
6	-6.667828000	-1.520947000	-0.797535000
1	-5.201743000	-2.544343000	-2.005481000
6	-6.860057000	-1.135409000	0.668833000
1	-5.678676000	0.668730000	0.505955000
1	-5.837646000	0.059742000	2.158342000
1	-7.042682000	-2.133044000	2.576118000
1	-7.717841000	-3.039577000	1.219139000
1	-7.806315000	-0.594084000	0.783736000
1	-6.661993000	-0.623670000	-1.420395000
1	-7.495596000	-2.153116000	-1.127118000
8	-3.296824000	-0.184260000	1.412318000
1	-3.155197000	0.529929000	0.751318000
6	-2.914205000	3.073121000	-0.309055000
6	-4.332096000	3.365190000	0.186221000
6	-4.492082000	4.864133000	0.431125000
1	-4.510881000	2.808465000	1.108318000
1	-5.050074000	3.022024000	-0.561768000
6	-2.660507000	3.844166000	-1.608146000
6	-2.825178000	5.342082000	-1.361257000
1	-3.367333000	3.505167000	-2.369455000
1	-1.650770000	3.622432000	-1.961504000
6	-4.244534000	5.625935000	-0.870507000
1	-4.969223000	5.315703000	-1.626714000
1	-4.372692000	6.697839000	-0.703718000
6	-1.908588000	3.533658000	0.751093000
6	-3.486278000	5.318707000	1.488260000
1	-5.509768000	5.064821000	0.784440000
6	-1.819365000	5.797763000	-0.305245000
1	-2.645511000	5.885946000	-2.295757000
6	-2.068171000	5.033160000	0.994476000
1	-0.897071000	3.302059000	0.409221000
1	-2.085083000	2.982741000	1.677853000
1	-3.667430000	4.788634000	2.425978000
1	-3.605141000	6.387641000	1.678946000
1	-1.347382000	5.356625000	1.754981000
1	-0.803092000	5.615562000	-0.659916000
1	-1.924286000	6.870851000	-0.129590000
8	-2.811925000	1.676051000	-0.532559000
1	-1.922202000	1.487538000	-0.896854000
6	2.781689000	3.006402000	-0.543264000
6	2.541834000	3.066508000	0.965364000
6	2.950142000	4.437181000	1.500045000
1	1.483936000	2.882207000	1.164284000
1	3.116126000	2.277586000	1.456555000
6	1.949202000	4.092256000	-1.230429000
6	2.347319000	5.464860000	-0.691074000
1	2.115114000	4.044715000	-2.308575000
1	0.890254000	3.900530000	-1.047665000
6	2.110100000	5.516207000	0.818062000
1	1.052721000	5.354487000	1.036330000
1	2.384622000	6.501149000	1.202154000
6	4.264559000	3.265907000	-0.838068000

6	4.430625000	4.680689000	1.209654000
1	2.779700000	4.469146000	2.582124000
6	3.826258000	5.718047000	-0.980645000
1	1.742574000	6.235455000	-1.183074000
6	4.665577000	4.637574000	-0.300157000
1	4.429414000	3.213654000	-1.916793000
1	4.872822000	2.488789000	-0.370041000
1	5.036435000	3.917062000	1.703058000
1	4.731835000	5.653659000	1.604071000
1	5.727046000	4.812439000	-0.510339000
1	4.003214000	5.702922000	-2.058480000
1	4.114960000	6.703134000	-0.607480000
8	2.379456000	1.749290000	-1.066695000
1	2.640184000	1.034457000	-0.445431000
6	-0.216904000	0.671237000	-2.902162000
6	0.141706000	1.699416000	-3.982529000
6	0.176626000	1.018557000	-5.349054000
1	1.116111000	2.134200000	-3.748481000
1	-0.598220000	2.503523000	-3.979847000
6	-1.585628000	0.064710000	-3.213979000
6	-1.547616000	-0.612628000	-4.582399000
1	-2.343139000	0.850322000	-3.201628000
1	-1.839170000	-0.656835000	-2.435875000
6	-1.195455000	0.418223000	-5.654468000
1	-1.950647000	1.207354000	-5.673720000
1	-1.182595000	-0.057224000	-6.638083000
6	0.841699000	-0.434101000	-2.902521000
6	1.229191000	-0.089391000	-5.341361000
1	0.430387000	1.758494000	-6.117305000
6	-0.495628000	-1.720624000	-4.578470000
1	-2.531997000	-1.043728000	-4.798627000
6	0.873473000	-1.117592000	-4.267885000
1	0.613858000	-1.148451000	-2.109429000
1	1.814902000	0.009871000	-2.683705000
1	2.214132000	0.335523000	-5.135334000
1	1.269318000	-0.570708000	-6.321508000
1	1.630817000	-1.910894000	-4.261146000
1	-0.472924000	-2.216225000	-5.552226000
1	-0.751933000	-2.471960000	-3.828227000
8	-0.288131000	1.293939000	-1.627681000
1	0.631134000	1.509614000	-1.364641000
6	0.443537000	-3.661870000	-0.330208000
6	0.574540000	-4.561804000	0.904431000
6	0.200473000	-5.997031000	0.539192000
1	-0.084528000	-4.180775000	1.687234000
1	1.599369000	-4.525357000	1.280938000
6	1.366281000	-4.175174000	-1.437099000
6	0.987487000	-5.610344000	-1.796548000
1	2.402590000	-4.136238000	-1.094681000
1	1.277863000	-3.526359000	-2.310371000
6	1.131118000	-6.502032000	-0.563417000
1	2.165771000	-6.488195000	-0.212307000
1	0.878784000	-7.533412000	-0.819990000
6	-1.009065000	-3.717465000	-0.813694000
6	-1.245483000	-6.042126000	0.046657000

1	0.304647000	-6.634353000	1.424804000
6	-0.457804000	-5.651683000	-2.290772000
1	1.654688000	-5.970295000	-2.588256000
6	-1.385227000	-5.148909000	-1.185135000
1	-1.128545000	-3.055988000	-1.674877000
1	-1.652526000	-3.352383000	-0.010165000
1	-1.917228000	-5.695801000	0.835524000
1	-1.522342000	-7.068917000	-0.203194000
1	-2.422258000	-5.172163000	-1.540899000
1	-0.727112000	-6.673952000	-2.565755000
1	-0.564362000	-5.026839000	-3.180651000
8	0.806642000	-2.323540000	-0.025296000
1	0.171071000	-2.007663000	0.656913000
6	4.098965000	-1.106583000	0.437627000
6	5.179814000	-0.239770000	1.085038000
6	6.539648000	-0.919442000	0.935291000
1	4.938917000	-0.092212000	2.140180000
1	5.195756000	0.739777000	0.604737000
6	4.422240000	-1.306333000	-1.047036000
6	5.782098000	-1.985895000	-1.190077000
1	4.428983000	-0.335767000	-1.548605000
1	3.638790000	-1.913909000	-1.505691000
6	6.857395000	-1.109498000	-0.547883000
1	6.893336000	-0.139327000	-1.048843000
1	7.836726000	-1.580093000	-0.659158000
6	4.076530000	-2.466682000	1.139435000
6	6.512987000	-2.280939000	1.628603000
1	7.308805000	-0.289305000	1.396246000
6	5.748478000	-3.346627000	-0.495292000
1	6.009393000	-2.123624000	-2.253166000
6	5.432957000	-3.151399000	0.987246000
1	3.287090000	-3.078957000	0.701174000
1	3.844845000	-2.322611000	2.197591000
1	6.304193000	-2.152839000	2.693151000
1	7.487453000	-2.765023000	1.533786000
1	5.404526000	-4.126489000	1.487145000
1	4.989702000	-3.981489000	-0.958934000
1	6.713809000	-3.845471000	-0.606619000
8	2.849262000	-0.443714000	0.583403000
1	2.153049000	-1.058733000	0.281065000
6	-0.421040000	-0.933473000	2.992603000
6	-1.401035000	-0.831946000	4.165454000
6	-0.737866000	-0.094945000	5.327070000
1	-1.698746000	-1.837283000	4.473177000
1	-2.296705000	-0.301587000	3.834271000
6	-0.021556000	0.476053000	2.545965000
6	0.636105000	1.213999000	3.709643000
1	0.671793000	0.404733000	1.705880000
1	-0.914843000	1.005432000	2.209155000
6	-0.342926000	1.311007000	4.878625000
1	-1.232441000	1.866353000	4.572336000
1	0.119707000	1.850241000	5.708786000
6	0.826253000	-1.696066000	3.439962000
6	0.511717000	-0.855156000	5.770553000
1	-1.441782000	-0.028672000	6.164853000

6	1.886365000	0.448460000	4.140043000
1	0.919286000	2.223227000	3.391300000
6	1.487963000	-0.953634000	4.598864000
1	1.516822000	-1.774176000	2.600350000
1	0.543510000	-2.705702000	3.745398000
1	0.237209000	-1.856461000	6.110647000
1	0.985257000	-0.335591000	6.607042000
1	2.382135000	-1.503444000	4.916034000
1	2.575427000	0.377209000	3.296104000
1	2.391106000	0.978306000	4.951462000
8	-1.014555000	-1.642292000	1.913438000
1	-1.829715000	-1.149668000	1.668398000

**2bCL4**			
6	2.777079000	0.282455000	-1.125878000
6	1.930039000	0.140053000	-2.395012000
6	2.809147000	0.283245000	-3.632873000
6	3.470059000	1.659797000	-3.624408000
6	4.321380000	1.803935000	-2.364736000
1	2.704686000	2.439249000	-3.646987000
1	4.097231000	1.779179000	-4.511090000
6	3.453862000	1.661284000	-1.112116000
1	1.152868000	0.907882000	-2.385661000
1	1.439719000	-0.836057000	-2.390261000
6	5.407991000	0.726102000	-2.336937000
1	4.794758000	2.793327000	-2.358453000
6	3.884547000	-0.801256000	-3.612823000
1	2.193567000	0.175435000	-4.533048000
6	3.870180000	-0.792889000	-1.099263000
6	4.735522000	-0.648938000	-2.353578000
1	5.507019000	-1.427987000	-2.341376000
1	3.417139000	-1.788688000	-3.627783000
1	4.517413000	-0.715983000	-4.499375000
8	1.951557000	0.112120000	0.018139000
6	4.310035000	1.813500000	0.144202000
1	2.670934000	2.429561000	-1.117726000
6	6.269833000	0.877813000	-1.085168000
1	6.040054000	0.828714000	-3.227208000
6	4.727401000	-0.639225000	0.156219000
1	3.391684000	-1.778449000	-1.094363000
6	5.391960000	0.735573000	0.156161000
1	6.757232000	1.855976000	-1.087234000
1	7.051454000	0.114090000	-1.078707000
1	5.485597000	-1.425666000	0.182750000
1	4.095599000	-0.742488000	1.040140000
1	3.679735000	1.712104000	1.029570000
1	4.766775000	2.806249000	0.162433000
1	6.007757000	0.844701000	1.056146000
1	1.281986000	0.832497000	0.005871000
6	0.299392000	-2.686989000	1.093742000
6	0.218102000	-1.787085000	2.331740000
6	0.374364000	-2.621334000	3.598730000
6	-0.741477000	-3.662956000	3.653533000
6	-0.652790000	-4.566532000	2.425231000
1	-1.713768000	-3.165209000	3.678938000

1	-0.647720000	-4.263204000	4.561634000
6	-0.808606000	-3.746481000	1.142225000
1	-0.745809000	-1.272715000	2.336699000
1	1.005607000	-1.031769000	2.271289000
6	0.700379000	-5.281664000	2.395607000
1	-1.454777000	-5.313179000	2.466177000
6	1.730080000	-3.323756000	3.576218000
1	0.312109000	-1.967964000	4.476308000
6	1.656576000	-3.406478000	1.067775000
6	1.810544000	-4.228876000	2.348812000
1	2.784874000	-4.731959000	2.332831000
1	2.532105000	-2.582551000	3.543893000
1	1.858081000	-3.918141000	4.484015000
8	0.122156000	-1.902984000	-0.077971000
6	-0.719468000	-4.656936000	-0.081588000
1	-1.779096000	-3.238171000	1.147073000
6	0.788867000	-6.195554000	1.175039000
1	0.810540000	-5.881849000	3.306775000
6	1.743263000	-4.314378000	-0.158110000
1	2.449218000	-2.650782000	1.019379000
6	0.634518000	-5.362885000	-0.095594000
1	0.002864000	-6.953396000	1.221532000
1	1.752112000	-6.711711000	1.167818000
1	2.721802000	-4.799745000	-0.190764000
1	1.629462000	-3.715096000	-1.064140000
1	-0.831490000	-4.057636000	-0.986949000
1	-1.528445000	-5.391049000	-0.054231000
1	0.697883000	-6.016540000	-0.973134000
1	0.847709000	-1.234786000	-0.092260000
6	-2.755910000	-0.308290000	-1.127448000
6	-1.896373000	-0.274983000	-2.395437000
6	-2.773250000	-0.451621000	-3.630494000
6	-3.495110000	-1.794828000	-3.548064000
6	-4.360994000	-1.827749000	-2.290449000
1	-2.765552000	-2.607532000	-3.517580000
1	-4.120578000	-1.937855000	-4.432459000
6	-3.495614000	-1.652299000	-1.040849000
1	-1.152265000	-1.072765000	-2.336337000
1	-1.364892000	0.678424000	-2.440643000
6	-5.397892000	-0.702715000	-2.334441000
1	-4.878069000	-2.793065000	-2.231725000
6	-3.799522000	0.678417000	-3.682998000
1	-2.147963000	-0.423507000	-4.530001000
6	-3.800414000	0.814071000	-1.172549000
6	-4.663703000	0.637485000	-2.424155000
1	-5.399495000	1.449808000	-2.463128000
1	-3.288326000	1.641704000	-3.752240000
1	-4.430609000	0.569392000	-4.568200000
8	-1.931422000	-0.111357000	0.011599000
6	-4.364129000	-1.690127000	0.215593000
1	-2.751596000	-2.455983000	-0.994339000
6	-6.273278000	-0.743598000	-1.083567000
1	-6.028490000	-0.827590000	-3.222897000
6	-4.671906000	0.773812000	0.081907000
1	-3.276393000	1.776043000	-1.222814000

6	-5.397788000	-0.567629000	0.155038000
1	-6.802937000	-1.698166000	-1.033429000
1	-7.020782000	0.052431000	-1.127808000
1	-5.394797000	1.593170000	0.054967000
1	-4.042048000	0.899583000	0.964007000
1	-3.733809000	-1.558645000	1.097746000
1	-4.861993000	-2.659840000	0.292396000
1	-6.023499000	-0.596280000	1.054372000
1	-1.250454000	-0.825222000	0.006683000
6	-0.365625000	3.135386000	0.536455000
6	-0.567222000	4.199588000	-0.552869000
6	-0.817536000	5.565267000	0.074809000
6	-2.071883000	5.492545000	0.942354000
6	-1.869274000	4.446168000	2.035910000
1	-2.935129000	5.225920000	0.327614000
1	-2.270541000	6.467037000	1.394658000
6	-1.612339000	3.064525000	1.425806000
1	-1.412659000	3.906566000	-1.180995000
1	0.322981000	4.225287000	-1.185881000
6	-0.675145000	4.829385000	2.913536000
1	-2.771686000	4.393837000	2.656773000
6	0.383091000	5.947704000	0.937367000
1	-0.957344000	6.311841000	-0.715262000
6	0.830193000	3.519297000	1.423648000
6	0.574612000	4.900654000	2.032547000
1	1.436706000	5.178310000	2.650779000
1	1.281645000	6.006849000	0.318529000
1	0.220825000	6.929603000	1.388296000
8	-0.091459000	1.899741000	-0.113870000
6	-1.425026000	2.034327000	2.539210000
1	-2.470056000	2.775957000	0.807201000
6	-0.482669000	3.799474000	4.024839000
1	-0.857816000	5.814298000	3.359778000
6	1.024157000	2.487821000	2.535177000
1	1.730677000	3.554156000	0.800652000
6	-0.229856000	2.427346000	3.403981000
1	-1.373569000	3.765810000	4.656860000
1	0.363428000	4.087170000	4.653779000
1	1.887365000	2.769284000	3.143859000
1	1.221692000	1.506739000	2.101748000
1	-1.268752000	1.045012000	2.107412000
1	-2.330699000	1.993400000	3.149728000
1	-0.090771000	1.683723000	4.196788000
1	-0.868403000	1.300477000	-0.055116000

**3bCL7**			
6	-0.853712000	2.567146000	-0.326882000
6	-2.091836000	2.759430000	0.545833000
6	-2.616843000	4.183503000	0.383765000
6	-1.549606000	5.194821000	0.808977000
6	-0.307538000	4.991670000	-0.062222000
1	-1.284764000	5.022361000	1.859251000
6	-2.079465000	6.618304000	0.649693000
6	0.219853000	3.567789000	0.101741000
1	-1.835382000	2.568101000	1.589648000

1	-2.854708000	2.046062000	0.234494000
6	-0.680831000	5.255146000	-1.522787000
1	0.471360000	5.699481000	0.247057000
6	-2.994661000	4.443662000	-1.076162000
1	-3.503864000	4.308998000	1.015780000
6	-1.238860000	2.816991000	-1.786182000
6	-1.750450000	4.246023000	-1.944332000
1	-2.013622000	4.414293000	-2.994496000
1	-3.757644000	3.722218000	-1.386794000
6	-3.527160000	5.865573000	-1.236034000
8	-0.395975000	1.223492000	-0.142399000
1	1.116668000	3.426018000	-0.506356000
1	0.488605000	3.385644000	1.144178000
1	0.208273000	5.127911000	-2.153198000
6	-1.211062000	6.678412000	-1.684180000
1	-0.375092000	2.647947000	-2.432091000
1	-2.029285000	2.123678000	-2.075361000
6	-2.450841000	6.863403000	-0.811412000
1	-1.462401000	6.861719000	-2.731500000
1	-0.440747000	7.397348000	-1.393642000
1	-4.420775000	6.000031000	-0.621661000
1	-3.807684000	6.040943000	-2.277119000
1	-1.317877000	7.335485000	0.966122000
1	-2.956644000	6.759448000	1.285656000
1	0.446210000	1.103589000	-0.629710000
1	-2.830307000	7.885080000	-0.926644000
6	-4.354601000	-3.808997000	4.242488000
6	-4.670434000	-3.353039000	2.819341000
6	-3.639806000	-2.316992000	2.376557000
6	-3.678204000	-1.104313000	3.309425000
6	-3.376054000	-1.568437000	4.736339000
1	-4.678125000	-0.655785000	3.277965000
6	-2.652218000	-0.065107000	2.865525000
6	-4.404528000	-2.605934000	5.181936000
1	-5.672977000	-2.919238000	2.782945000
1	-4.651330000	-4.209503000	2.140841000
6	-1.968677000	-2.170209000	4.770314000
1	-3.415941000	-0.706033000	5.412436000
6	-2.233178000	-2.917960000	2.412825000
1	-3.861990000	-1.994584000	1.352467000
6	-2.956741000	-4.423392000	4.281943000
6	-1.929177000	-3.383885000	3.838288000
1	-0.927285000	-3.828589000	3.866860000
1	-2.190292000	-3.778970000	1.734997000
6	-1.210966000	-1.877260000	1.963416000
1	-5.092076000	-4.554325000	4.561247000
1	-4.191644000	-2.923505000	6.205600000
1	-5.404276000	-2.165041000	5.169188000
1	-1.737575000	-2.491391000	5.792744000
6	-0.937408000	-1.133653000	4.330141000
1	-2.728023000	-4.762317000	5.295273000
1	-2.913321000	-5.292277000	3.620674000
6	-1.249629000	-0.674243000	2.905594000
1	0.067176000	-1.560945000	4.360492000
1	-0.956521000	-0.270147000	4.998664000

1	-1.444214000	-1.543822000	0.950940000
1	-0.207768000	-2.307054000	1.965186000
1	-2.690408000	0.808676000	3.520455000
1	-2.867866000	0.262128000	1.847276000
8	-0.267496000	0.288131000	2.538785000
1	-0.414385000	0.563599000	1.611575000
6	-9.686437000	-0.194501000	0.497453000
6	-8.659990000	-1.210784000	0.993880000
6	-7.293468000	-0.889621000	0.392685000
6	-7.360241000	-0.948761000	-1.135088000
6	-8.399531000	0.062493000	-1.624511000
1	-7.662649000	-1.956586000	-1.444676000
6	-5.992731000	-0.631200000	-1.733076000
6	-9.767877000	-0.258384000	-1.026328000
1	-8.964592000	-2.219667000	0.704745000
1	-8.602285000	-1.178399000	2.084948000
6	-7.954558000	1.466403000	-1.206824000
1	-8.462226000	0.014666000	-2.718099000
6	-6.850019000	0.514256000	0.808909000
1	-6.558420000	-1.620940000	0.749525000
6	-9.257119000	1.208374000	0.921938000
6	-7.889242000	1.526600000	0.321477000
1	-7.583599000	2.533546000	0.629764000
1	-6.783376000	0.563382000	1.902911000
6	-5.484063000	0.835873000	0.210739000
1	-10.668068000	-0.424178000	0.927616000
1	-10.508601000	0.458210000	-1.389501000
1	-10.084870000	-1.255886000	-1.340651000
1	-8.686753000	2.199238000	-1.566119000
6	-6.589787000	1.790779000	-1.808535000
1	-9.991716000	1.941600000	0.580222000
1	-9.206342000	1.268413000	2.012022000
6	-5.553462000	0.777736000	-1.319929000
1	-6.271506000	2.794160000	-1.517975000
1	-6.632286000	1.758181000	-2.898852000
1	-4.741133000	0.115780000	0.561172000
1	-5.166666000	1.834380000	0.520375000
1	-6.034136000	-0.689410000	-2.823459000
1	-5.251771000	-1.354008000	-1.382345000
8	-4.310470000	1.115693000	-1.900596000
1	-3.656946000	0.430433000	-1.652512000
6	0.144708000	-3.920839000	-5.637547000
6	1.251278000	-3.099493000	-4.978153000
6	0.655190000	-2.258541000	-3.852039000
6	-0.411703000	-1.310885000	-4.404013000
6	-1.509770000	-2.138766000	-5.074791000
1	0.044596000	-0.646772000	-5.148076000
6	-0.992405000	-0.465996000	-3.272599000
6	-0.917706000	-2.980530000	-6.203740000
1	1.723168000	-2.449086000	-5.718954000
1	2.021290000	-3.764150000	-4.577940000
6	-2.153832000	-3.043069000	-4.021240000
1	-2.270617000	-1.464756000	-5.485264000
6	0.009604000	-3.164637000	-2.802918000
1	1.444425000	-1.667359000	-3.374485000

6	-0.496062000	-4.837537000	-4.597337000
6	-1.088865000	-3.994085000	-3.470296000
1	-1.546079000	-4.655811000	-2.724726000
1	0.769241000	-3.841786000	-2.393705000
6	-0.572294000	-2.323134000	-1.671189000
1	0.568999000	-4.525258000	-6.447349000
1	-1.707510000	-3.560456000	-6.687302000
1	-0.472723000	-2.327252000	-6.958295000
1	-2.955684000	-3.627885000	-4.486949000
6	-2.744639000	-2.199008000	-2.894682000
1	-1.280960000	-5.437839000	-5.063643000
1	0.253197000	-5.522552000	-4.192352000
6	-1.640542000	-1.377407000	-2.228599000
1	-3.216605000	-2.839475000	-2.146169000
1	-3.503921000	-1.521748000	-3.289520000
1	0.215271000	-1.731611000	-1.196988000
1	-1.022522000	-2.971385000	-0.915511000
1	-1.740355000	0.227798000	-3.662938000
1	-0.193196000	0.110362000	-2.801342000
8	-2.235721000	-0.622312000	-1.180073000
1	-1.573296000	0.016714000	-0.842390000
6	4.417594000	-7.152230000	0.462693000
6	5.611988000	-6.395591000	-0.114543000
6	5.295248000	-4.902271000	-0.161120000
6	4.068647000	-4.647998000	-1.041243000
6	2.880288000	-5.420095000	-0.464021000
1	4.272876000	-5.004910000	-2.057894000
6	3.763758000	-3.153026000	-1.093357000
6	3.193743000	-6.914091000	-0.419228000
1	5.832100000	-6.759182000	-1.121269000
1	6.496095000	-6.567501000	0.504123000
6	2.592249000	-4.896747000	0.944799000
1	1.999207000	-5.255847000	-1.095957000
6	5.005246000	-4.378881000	1.247927000
1	6.155167000	-4.363330000	-0.576636000
6	4.131344000	-6.645199000	1.874639000
6	3.817123000	-5.151385000	1.826119000
1	3.612034000	-4.790581000	2.840890000
1	5.885299000	-4.541516000	1.881537000
6	4.690109000	-2.885749000	1.205360000
1	4.643181000	-8.224089000	0.495961000
1	2.335361000	-7.460197000	-0.020557000
1	3.385584000	-7.283336000	-1.429732000
1	1.729666000	-5.431706000	1.359942000
6	2.277891000	-3.404304000	0.895902000
1	3.284161000	-7.188221000	2.300600000
1	4.998353000	-6.821046000	2.515966000
6	3.468236000	-2.637980000	0.314825000
1	2.076164000	-3.026269000	1.901385000
1	1.396788000	-3.226394000	0.276424000
1	5.544067000	-2.328030000	0.812880000
1	4.476935000	-2.516061000	2.210547000
1	2.905005000	-2.956743000	-1.737525000
1	4.622560000	-2.615823000	-1.501756000
8	3.180506000	-1.248441000	0.236446000

1	2.906124000	-0.961517000	1.138018000
6	6.445428000	3.398868000	-4.352626000
6	5.563284000	2.678038000	-5.369856000
6	4.435844000	1.949657000	-4.641424000
6	5.012695000	0.924776000	-3.662109000
6	5.908819000	1.650704000	-2.656074000
1	5.610446000	0.191443000	-4.216197000
6	3.883218000	0.199913000	-2.935729000
6	7.037784000	2.378527000	-3.382710000
1	6.160232000	1.961047000	-5.938512000
1	5.143506000	3.398693000	-6.075759000
6	5.054562000	2.649415000	-1.872475000
1	6.337142000	0.919163000	-1.960568000
6	3.581875000	2.948105000	-3.856999000
1	3.804946000	1.433047000	-5.374438000
6	5.601901000	4.407096000	-3.575147000
6	4.474716000	3.676321000	-2.848708000
1	3.872153000	4.404184000	-2.291631000
1	3.150070000	3.678854000	-4.550972000
6	2.448964000	2.225410000	-3.132428000
1	7.254571000	3.922298000	-4.874084000
1	7.680163000	2.883594000	-2.657298000
1	7.651391000	1.658280000	-3.929102000
1	5.683385000	3.165486000	-1.136853000
6	3.927769000	1.922525000	-1.143548000
1	6.226696000	4.936043000	-2.851447000
1	5.182061000	5.147911000	-4.259801000
6	3.030917000	1.203847000	-2.155997000
1	3.332979000	2.639385000	-0.571580000
1	4.336500000	1.184925000	-0.448062000
1	1.802325000	1.711721000	-3.847398000
1	1.841032000	2.948560000	-2.584978000
1	4.290349000	-0.532463000	-2.236571000
1	3.253804000	-0.328815000	-3.655322000
8	1.954715000	0.544174000	-1.498604000
1	2.344624000	-0.152103000	-0.929253000
6	2.997959000	0.584101000	3.289496000
6	2.558761000	1.845743000	2.546287000
6	3.302062000	3.058906000	3.096051000
6	2.985389000	3.235082000	4.583129000
6	3.411666000	1.968045000	5.329382000
1	1.905593000	3.379804000	4.706691000
6	3.724816000	4.450066000	5.139488000
6	2.670535000	0.752674000	4.776144000
1	1.481889000	1.968908000	2.679298000
1	2.759089000	1.722547000	1.480999000
6	4.922918000	1.785966000	5.166239000
1	3.174188000	2.078890000	6.394076000
6	4.812371000	2.875573000	2.929971000
1	2.984630000	3.955354000	2.549475000
6	4.505353000	0.393527000	3.123397000
6	5.239499000	1.610838000	3.678959000
1	6.318889000	1.464269000	3.554255000
1	5.046877000	2.761222000	1.865044000
6	5.554000000	4.090450000	3.483932000

8	2.340086000	-0.557284000	2.756030000
1	2.963887000	-0.150176000	5.316953000
1	1.591553000	0.879824000	4.891037000
1	5.239018000	0.888056000	5.710422000
6	5.664739000	2.999528000	5.722799000
1	4.815221000	-0.509233000	3.654066000
1	4.738971000	0.260208000	2.065236000
6	5.229588000	4.253583000	4.967565000
1	6.742690000	2.855576000	5.616106000
1	5.445846000	3.111520000	6.787443000
1	5.255018000	4.987933000	2.936859000
1	6.630734000	3.960212000	3.350838000
1	3.484859000	4.578080000	6.197744000
1	3.404280000	5.351869000	4.612062000
1	1.381442000	-0.340047000	2.715578000
1	5.761589000	5.124972000	5.365856000

**4bCL8**			
6	-3.838512000	2.887562000	-0.194337000
6	-2.663329000	1.938038000	-0.434884000
6	-1.389740000	2.738641000	-0.677180000
6	-1.554237000	3.632176000	-1.908644000
6	-2.735847000	4.577773000	-1.672853000
1	-1.761361000	3.006932000	-2.784352000
6	-0.281322000	4.438317000	-2.157717000
6	-4.012931000	3.775975000	-1.429608000
1	-2.889973000	1.308588000	-1.297349000
1	-2.537487000	1.293632000	0.436637000
6	-2.424611000	5.461746000	-0.461261000
1	-2.875040000	5.211107000	-2.557029000
6	-1.087697000	3.618787000	0.537651000
1	-0.555415000	2.046295000	-0.837582000
6	-3.540614000	3.766869000	1.019964000
6	-2.265000000	4.567596000	0.770815000
1	-2.057253000	5.192243000	1.646587000
1	-0.955908000	2.985000000	1.420707000
6	0.195414000	4.404029000	0.294864000
8	-5.022727000	2.150108000	0.070788000
1	-4.859635000	4.448876000	-1.275735000
1	-4.232682000	3.145531000	-2.294421000
1	-3.255152000	6.158050000	-0.299237000
6	-1.143757000	6.261554000	-0.698976000
1	-4.385997000	4.434548000	1.197299000
1	-3.419707000	3.133600000	1.900720000
6	0.023772000	5.297547000	-0.932047000
1	-0.924002000	6.891338000	0.165571000
1	-1.262971000	6.913974000	-1.568458000
1	1.018109000	3.709995000	0.124485000
1	0.446167000	5.016990000	1.161562000
1	-0.417493000	5.082795000	-3.032016000
1	0.565098000	3.780327000	-2.362933000
1	-5.160041000	1.541845000	-0.694316000
8	1.246856000	6.016038000	-1.110776000
1	1.294358000	6.310348000	-2.027562000
6	7.795127000	8.491404000	0.937020000

6	6.529552000	9.238495000	0.501386000
6	5.428921000	8.235023000	0.164663000
6	5.882363000	7.314325000	-0.971190000
6	7.149964000	6.576615000	-0.532285000
1	6.104440000	7.917325000	-1.858832000
6	4.780000000	6.313297000	-1.309237000
6	8.253266000	7.575769000	-0.197759000
1	6.760137000	9.857091000	-0.368792000
1	6.197485000	9.901064000	1.306486000
6	6.821469000	5.721341000	0.694031000
1	7.490454000	5.927037000	-1.346574000
6	5.099770000	7.379099000	1.390773000
1	4.530097000	8.779172000	-0.147762000
6	7.469471000	7.641564000	2.170397000
6	6.367236000	6.641045000	1.830354000
1	6.142421000	6.036983000	2.716504000
1	4.758191000	8.028455000	2.204983000
6	3.997227000	6.377879000	1.056427000
8	8.857993000	9.392020000	1.199840000
1	9.162588000	7.058049000	0.111445000
1	8.497723000	8.186913000	-1.067929000
1	7.720114000	5.177632000	1.006294000
6	5.721797000	4.716217000	0.362846000
1	8.372216000	7.118564000	2.493699000
1	7.145258000	8.290539000	2.989585000
6	4.462829000	5.461503000	-0.076445000
1	5.490268000	4.110852000	1.239998000
1	6.047394000	4.047291000	-0.437226000
1	3.090874000	6.901586000	0.741981000
1	3.752252000	5.774566000	1.932934000
1	5.097425000	5.667618000	-2.131356000
1	3.876345000	6.840924000	-1.623589000
1	8.592931000	9.979096000	1.916552000
8	3.457368000	4.497856000	-0.379362000
1	2.629492000	5.000249000	-0.535562000
6	-5.346825000	0.082304000	2.879618000
6	-5.067059000	-1.173704000	3.704557000
6	-6.051267000	-1.252625000	4.868236000
6	-5.911381000	-0.022286000	5.768687000
6	-6.187649000	1.232112000	4.935769000
1	-4.889437000	0.022774000	6.162852000
6	-6.893616000	-0.108264000	6.934594000
6	-5.204310000	1.318985000	3.771727000
1	-4.039698000	-1.140590000	4.073179000
1	-5.168712000	-2.052455000	3.064902000
6	-7.623580000	1.168237000	4.409074000
1	-6.074104000	2.118772000	5.569755000
6	-7.487306000	-1.316138000	4.340841000
1	-5.839441000	-2.155553000	5.452924000
6	-6.778826000	0.022934000	2.343354000
6	-7.762448000	-0.061446000	3.507718000
1	-8.783653000	-0.106847000	3.113147000
1	-7.600121000	-2.202940000	3.706671000
6	-8.472379000	-1.404361000	5.504250000
8	-4.409947000	0.126081000	1.814705000

1	-5.403260000	2.213605000	3.176621000
1	-4.178847000	1.378910000	4.144414000
1	-7.834224000	2.070418000	3.824206000
6	-8.608610000	1.085903000	5.571227000
1	-6.973530000	0.917116000	1.746781000
1	-6.885140000	-0.848724000	1.694289000
6	-8.328490000	-0.168095000	6.398762000
1	-9.635543000	1.045060000	5.204944000
1	-8.519709000	1.961630000	6.216004000
1	-8.278872000	-2.307858000	6.090733000
1	-9.497346000	-1.459528000	5.131119000
1	-6.789722000	0.763400000	7.584506000
1	-6.686487000	-1.000667000	7.533295000
1	-4.629662000	0.909682000	1.256754000
8	-9.271019000	-0.187815000	7.457726000
1	-9.116263000	-0.974647000	7.992093000
6	7.293549000	-8.582014000	1.125021000
6	7.123576000	-7.383041000	2.057490000
6	6.174835000	-6.368759000	1.425916000
6	6.739159000	-5.881301000	0.089201000
6	6.913518000	-7.083756000	-0.841999000
1	7.715499000	-5.410975000	0.257600000
6	5.794429000	-4.862662000	-0.542263000
6	7.862768000	-8.102109000	-0.214906000
1	8.102768000	-6.937417000	2.241262000
1	6.731801000	-7.732324000	3.013800000
6	5.542192000	-7.719011000	-1.088411000
1	7.333154000	-6.745789000	-1.797794000
6	4.805924000	-7.005816000	1.177925000
1	6.051607000	-5.516927000	2.102806000
6	5.926525000	-9.228750000	0.877910000
6	4.979961000	-8.208760000	0.248603000
1	4.004856000	-8.677654000	0.077729000
1	4.388832000	-7.341206000	2.132949000
6	3.855907000	-5.986304000	0.557368000
8	8.182326000	-9.485312000	1.763202000
1	7.992606000	-8.955969000	-0.887165000
1	8.845234000	-7.654515000	-0.047532000
1	5.655686000	-8.571073000	-1.769351000
6	4.589936000	-6.703355000	-1.715474000
1	6.042451000	-10.092206000	0.215156000
1	5.522097000	-9.586074000	1.827409000
6	4.423417000	-5.503592000	-0.778007000
1	3.613408000	-7.159778000	-1.888395000
1	4.979742000	-6.363335000	-2.679672000
1	3.729139000	-5.141043000	1.234279000
1	2.878676000	-6.438457000	0.385037000
1	6.203733000	-4.511989000	-1.494220000
1	5.678770000	-3.999292000	0.115376000
1	8.284488000	-10.261679000	1.201594000
8	3.513568000	-4.559328000	-1.330007000
1	3.855514000	-4.276745000	-2.198531000
6	3.489796000	-2.543485000	3.327572000
6	4.505339000	-1.755124000	4.162890000
6	4.348241000	-0.260023000	3.891341000

6	4.579334000	0.032078000	2.406940000
6	3.560248000	-0.759146000	1.585573000
1	5.591762000	-0.284683000	2.129712000
6	4.427005000	1.523749000	2.127503000
6	3.722000000	-2.251159000	1.846768000
1	5.514880000	-2.083833000	3.905510000
1	4.346588000	-1.960802000	5.225781000
6	2.150511000	-0.300140000	1.965143000
1	3.730957000	-0.564220000	0.523449000
6	2.938855000	0.204662000	4.268518000
1	5.082916000	0.292057000	4.488668000
6	2.077632000	-2.092901000	3.715215000
6	1.922196000	-0.596122000	3.448844000
1	0.908800000	-0.285928000	3.728232000
1	2.770273000	0.019523000	5.335836000
6	2.787369000	1.700314000	3.996070000
8	3.656210000	-3.941125000	3.502307000
1	3.013619000	-2.828486000	1.250915000
1	4.729057000	-2.576478000	1.579824000
1	1.417213000	-0.855057000	1.368086000
6	1.988604000	1.193782000	1.696469000
1	1.348585000	-2.659309000	3.131242000
1	1.900872000	-2.305335000	4.774027000
6	3.017335000	1.987497000	2.510658000
1	0.985269000	1.521999000	1.975249000
1	2.124806000	1.398370000	0.633550000
1	3.507730000	2.270711000	4.584951000
1	1.788414000	2.042558000	4.271051000
1	4.600210000	1.726549000	1.066623000
1	5.159820000	2.092693000	2.704877000
1	3.496526000	-4.159344000	4.426870000
8	2.871131000	3.379746000	2.311801000
1	3.068851000	3.593614000	1.383260000
6	-6.489400000	0.150438000	-2.467739000
6	-6.554183000	-1.058536000	-3.405011000
6	-7.939906000	-1.147380000	-4.038474000
6	-9.005658000	-1.311123000	-2.951824000
6	-8.932981000	-0.108804000	-2.007031000
1	-8.806388000	-2.227713000	-2.385841000
6	-10.391936000	-1.405095000	-3.581946000
6	-7.547066000	-0.015244000	-1.374139000
1	-6.344197000	-1.964918000	-2.832480000
1	-5.787912000	-0.958153000	-4.177460000
6	-9.233945000	1.163129000	-2.804408000
1	-9.682410000	-0.224707000	-1.216105000
6	-8.239959000	0.124174000	-4.836324000
1	-7.973319000	-2.011906000	-4.711044000
6	-6.782311000	1.424186000	-3.260908000
6	-8.168580000	1.327215000	-3.891944000
1	-8.367081000	2.244766000	-4.458397000
1	-7.489956000	0.240662000	-5.627349000
6	-9.625896000	0.033969000	-5.470928000
8	-5.193804000	0.276297000	-1.902612000
1	-7.500826000	0.838437000	-0.694667000
1	-7.327748000	-0.918768000	-0.800958000

1	-9.199444000	2.027936000	-2.131822000
6	-10.621594000	1.075002000	-3.435539000
1	-6.731490000	2.284490000	-2.590198000
1	-6.019340000	1.551054000	-4.031868000
6	-10.686077000	-0.133167000	-4.376561000
1	-10.836905000	1.990141000	-3.995834000
1	-11.384869000	0.967809000	-2.661737000
1	-9.677123000	-0.817747000	-6.152746000
1	-9.832603000	0.940159000	-6.048768000
1	-11.157690000	-1.526844000	-2.814325000
1	-10.454017000	-2.263042000	-4.253161000
1	-5.029472000	-0.521535000	-1.344133000
8	-11.980943000	-0.280451000	-4.934915000
1	-12.189483000	0.512713000	-5.440860000
6	0.053400000	-5.241903000	-1.147269000
6	-1.164096000	-6.105706000	-0.813909000
6	-2.374168000	-5.215413000	-0.547651000
6	-2.094543000	-4.277953000	0.630120000
6	-0.873127000	-3.417130000	0.296526000
1	-1.881722000	-4.875236000	1.524073000
6	-3.308995000	-3.391713000	0.898428000
6	0.340156000	-4.304050000	0.030927000
1	-0.936130000	-6.716529000	0.061481000
1	-1.358693000	-6.780909000	-1.648814000
6	-1.193478000	-2.571668000	-0.938360000
1	-0.659143000	-2.752292000	1.142778000
6	-2.692097000	-4.370072000	-1.783606000
1	-3.238529000	-5.846138000	-0.309587000
6	-0.260855000	-4.393391000	-2.386722000
6	-1.472625000	-3.507015000	-2.117575000
1	-1.687952000	-2.907388000	-3.010105000
1	-2.907136000	-5.033376000	-2.628765000
6	-3.907530000	-3.484458000	-1.523986000
8	1.143273000	-6.102208000	-1.392940000
1	1.216827000	-3.695124000	-0.205254000
1	0.576796000	-4.900113000	0.915192000
1	-0.331426000	-1.936945000	-1.176716000
6	-2.407399000	-1.685492000	-0.670406000
1	0.609740000	-3.781815000	-2.634696000
1	-0.454158000	-5.057218000	-3.231887000
6	-3.623836000	-2.555169000	-0.344117000
1	-2.626298000	-1.073103000	-1.547597000
1	-2.209184000	-1.018361000	0.171232000
1	-4.785590000	-4.093196000	-1.298502000
1	-4.130686000	-2.882946000	-2.407647000
1	-3.109663000	-2.722030000	1.739003000
1	-4.177004000	-4.005759000	1.150384000
1	1.947251000	-5.550688000	-1.443147000
8	-4.780298000	-1.755966000	-0.134405000
1	-4.613019000	-1.164758000	0.636777000
6	3.343517000	2.424180000	-3.249127000
6	2.743259000	1.640401000	-2.079099000
6	3.094359000	0.162820000	-2.203049000
6	2.543014000	-0.402125000	-3.513821000
6	3.145895000	0.384104000	-4.681312000

1	1.453279000	-0.290048000	-3.526272000
6	2.891115000	-1.884253000	-3.622899000
6	2.793463000	1.866311000	-4.562078000
1	1.657885000	1.767512000	-2.077496000
1	3.138486000	2.055352000	-1.148239000
6	4.665233000	0.195458000	-4.663516000
1	2.744568000	-0.003327000	-5.624324000
6	4.614062000	-0.018180000	-2.184071000
1	2.649253000	-0.387742000	-1.366557000
6	4.866511000	2.244730000	-3.228852000
6	5.217210000	0.764438000	-3.353461000
1	6.308113000	0.652136000	-3.340356000
1	5.017496000	0.376768000	-1.244101000
6	4.968130000	-1.498434000	-2.295636000
8	2.994271000	3.791274000	-3.158102000
1	3.217008000	2.430485000	-5.394492000
1	1.710913000	2.004416000	-4.582662000
1	5.107542000	0.736210000	-5.507637000
6	5.016245000	-1.287588000	-4.782695000
1	5.305716000	2.812085000	-4.052274000
1	5.263078000	2.646771000	-2.292402000
6	4.408364000	-2.056255000	-3.607365000
1	6.103748000	-1.413193000	-4.784202000
1	4.628791000	-1.694175000	-5.719650000
1	4.542396000	-2.054987000	-1.458129000
1	6.054573000	-1.626715000	-2.271258000
1	2.491923000	-2.309991000	-4.544910000
1	2.465276000	-2.435383000	-2.782703000
1	3.272442000	4.098236000	-2.276492000
8	4.676312000	-3.453434000	-3.713914000
1	5.626812000	-3.572240000	-3.823078000
